# Clinical Pharmacology and Determinants of Response to UCART19, an Allogeneic Anti-CD19 CAR-T Cell Product, in Adult B-cell Acute Lymphoblastic Leukemia

**DOI:** 10.1158/2767-9764.CRC-22-0175

**Published:** 2022-11-30

**Authors:** Sandra Dupouy, Ibtissam Marchiq, Thibaud Derippe, Maria Almena-Carrasco, Agnieszka Jozwik, Sylvain Fouliard, Yasmina Adimy, Julia Geronimi, Charlotte Graham, Nitin Jain, Marcela V. Maus, Mohamad Mohty, Nicolas Boissel, Takanori Teshima, Koji Kato, Reuben Benjamin, Svetlana Balandraud

**Affiliations:** 1 Institut de Recherches Internationales Servier, Suresnes, France.; 2 Institut de Recherches Servier, Croissy-sur-Seine, France.; 3 Université Paris-Descartes, Paris, France.; 4 King's College Hospital NHS Foundation Trust, London, England.; 5 The University of Texas MD Anderson Cancer Center, Houston, Texas.; 6 Massachusetts General Hospital, Boston, Massachusetts.; 7 Sorbonne University, Saint-Antoine Hospital, INSERM UMRs 938, Paris, France.; 8 Saint-Louis Hospital, URP-3518, Institut de Recherche Saint-Louis, Paris, France.; 9 Hokkaido University Hospital, Sapporo, Japan.; 10 Department of Hematology, Oncology and Cardiovascular Medicine, Kyushu University Hospital, Fukuoka, Japan.; 11 King's College London, London, England.

## Abstract

**Background:**

UCART19^1^ is an “off-the-shelf” genome-edited anti-CD19 chimeric antigen receptor (CAR)-T cell product, manufactured from unrelated healthy donor cells.

**Methods:**

UCART19 was administered to 25 adult patients with relapsed or refractory (R/R) B-cell acute lymphoblastic leukemia (B-ALL) in the CALM trial. All patients underwent lymphodepletion with fludarabine and cyclophosphamide ± alemtuzumab and received one of three ascending doses of UCART19. Given the allogeneic nature of UCART19, we analyzed the impact of lymphodepletion, HLA disparities, and host immune system reconstitution on its kinetics, along with other factors known to affect autologous CAR-T cell clinical pharmacology.

**Results:**

Responder patients (12/25) had higher UCART19 expansion (*C*_max_) and exposure (AUCT_last_) than nonresponders (13/25), as measured by transgene levels in peripheral blood. The persistence of CAR^+^ T cells did not exceed 28 days in 10/25 patients and lasted beyond 42 days in 4/25. No significant correlation was found between UCART19 kinetics and administered cell dose, patient and product characteristics or HLA disparities. However, the number of prior lines of therapy and absence of alemtuzumab negatively impacted UCART19 expansion and persistence. Alemtuzumab exposure positively affected IL7 and UCART19 kinetics, while negatively correlating with host T lymphocyte AUC_0-28_.

**Conclusions:**

UCART19 expansion is a driver of response in adult patients with R/R B-ALL. These results shed light on the factors associated with UCART19 kinetics, which remain highly affected by the impact of alemtuzumab on IL7 and host-versus-graft rejection.

**Significance:**

First description of the clinical pharmacology of a genome-edited allogeneic anti-CD19 CAR-T cell product showing the crucial role of an alemtuzumab-based regimen in sustaining UCART19 expansion and persistence through increased IL7 availability and decreased host T lymphocyte population.

## Introduction

Over the past decade, autologous (patient-derived) chimeric antigen receptor (CAR)-T cell therapies have revolutionized the treatment of CD19^+^ B-cell malignancies. In addition to tisagenlecleucel (Kymriah) and axicabtagene ciloleucel (Yescarta) marketed for patients with relapsed or refractory (R/R) B-cell acute lymphoblastic leukemia (B-ALL), follicular lymphoma and/or large B-cell lymphoma (LBCL), respectively, three others were recently approved, brexucabtagene autoleucel (Tecartus) for R/R mantle cell lymphoma and adult B-ALL, lisocabtagene maraleucel (Breyanzi) and relmacabtagene autoleucel (Carteyva) for R/R LBCL, and many others are under active clinical development (1–8).

However, despite remarkable clinical outcomes, autologous CAR-T cell products face manufacturing, logistic and cost hurdles, thus limiting their use in patients with rapidly progressive disease or dysfunctional T cells (9–11). Allogeneic CAR-T cell therapies, in contrast, widen patient access to these innovative treatments through more easily scalable and readily available products with potentially reduced costs. To achieve these goals, genome-edited allogeneic T cells from healthy donors should overcome two major issues: graft-versus-host disease (GVHD), which can be life threatening, and host-versus-graft rejection of HLA-incompatible CAR-T cells, a major limiting factor of their expansion and persistence (11).

UCART19 is a first-in-class “off-the-shelf” allogeneic CAR-T cell immunotherapy. T cells from unrelated healthy donors were genetically engineered to express an anti-CD19 (murine 4G7 scFv)/4-1BB/CD3ζ CAR together with an RQR8 safety switch (CD20 mimotope; ref. 12). Cells were further genome-edited via transcription activator-like effector nucleases to simultaneously disrupt T-cell receptor alpha chain (*TRAC)* and *CD52* genes. Elimination of *TRAC* prevents GVHD, while the *CD52* gene knockout protects donor cells from early rejection through alemtuzumab, a powerful anti-CD52 peripheral lymphodepleting agent (13). We recently demonstrated the feasibility of administering UCART19 to adult and pediatric patients diagnosed with R/R B-ALL (14, 15). UCART19 induced preliminary antileukemic activity in these heavily pretreated populations and exhibited a manageable safety profile with minimal GVHD and moderate cytokine release syndromes.

Unlike conventional drugs, CAR-T cells exhibit unique pharmacodynamic and pharmacokinetic features. Clinical data of autologous second-generation anti-CD19 CAR-T cell therapies have shown that these “living” drugs distribute widely in tissues after intravenous administration, proliferate following activation and decline in number at a variable rate after exerting their antitumor effects. Parameters such as maximum CAR-T cell level (*C*_max_) and CAR-T cell exposure between the time of CAR-T cell administration (day 0) and day 28 (AUC_0-28_) have been associated with clinical response (16–18). Several factors, including lymphodepleting chemotherapy (19, 20), tumor burden (21, 22), targeted antigen expression level (23), CAR design, and CAR-T cell fitness (24–27) have been reported to influence CAR-T cell pharmacokinetic and therapeutic efficacy.

We have previously reported preliminary UCART19 PK results with a peak expansion similar to autologous CAR-T cells, around 14 days after UCART19 administration, and a variable persistence following a fludarabine and/or cyclophosphamide-based lymphodepletion regimen with or without alemtuzumab (14, 15). The current work goes deeper into the cellular kinetics and factors impacting expansion, persistence, and response of this genome-edited allogeneic anti-CD19 CAR-T cell therapy administered to 25 non–HLA-matched adult patients with R/R B-ALL as part of the CALM clinical trial.

## Materials and Methods

### Study Design

Twenty-five adult patients with CD19-positive R/R B-ALL were enrolled from August 2016 to July 2020 in an open-label nonrandomized phase I/II study conducted in eight clinical centers across Europe, USA, and Japan (NCT02746952). The CALM study comprised two phases: a dose escalation investigating three dose levels of UCART19 (6 × 10^6^, 6–8 × 10^7^, or 1.8–2.4 × 10^8^ total CAR^+^ cells) followed by a dose expansion at the recommended dose (6–8 × 10^7^ total CAR^+^ cells). All patients received a 6-day lymphodepletion regimen prior to UCART19 infusion (day 0) consisting of fludarabine (F) 30 mg/m^2^/day i.v. for 3 days (day-7 to day-5) and cyclophosphamide (C) 500 mg/m^2^/day i.v. for 3 days (day-4 to day-2), with or without alemtuzumab (A) 1 mg/kg, 40 or 60 mg flat doses (day-7 to day-3). The dose of alemtuzumab was modified during the trial to balance the infectious complications related to alemtuzumab use and UCART19 efficacy. An allogeneic stem cell transplantation (allo-SCT) could be performed at any time following disease evaluation at day 28 after UCART19 infusion. Nine GMP batches originating from 6 different donors were administered. Study design (Supplementary Fig. S1), study primary objectives and key inclusion and exclusion criteria were previously detailed by Benjamin and colleagues (15). Characteristics of the patient population and overall results are described in Supplementary Materials and Methods. Representativeness of the study population is discussed in Supplementary Table S1. Responder patients were defined as those achieving morphologic complete remission and/or complete remission with incomplete hematologic recovery (CR/CRi), minimal residual disease (MRD)-negative CR/CRi, or MRD indeterminate CR/CRi while nonresponders were those with relapsed, refractory, or progressive disease. UCART19 expansion was defined as two consecutive transgene concentrations above the limit of quantification by qPCR and confirmed by flow cytometry. The CALM study was conducted in accordance with the Declaration of Helsinki, International Conference on Harmonization, and Good Clinical Practice Guidelines and was approved by Institutional Review Boards/Ethics Committees. Written informed consent was obtained from all patients prior to inclusion in the study.

### UCART19 Manufacturing Process

Peripheral blood mononuclear cells (PBMC) from a healthy donor were collected by leukapheresis and frozen. Once thawed, cells were treated with benzonase to avoid clumping and then underwent T-cell activation. Activated T cells were subsequently transduced with a recombinant lentiviral vector expressing the anti-CD19 CAR construct, before being electroporated with transcription activator-like effector nucleases (TALEN®) mRNA to disrupt *TRAC* and *CD52* genes. Cells were then expanded in a bioreactor for 10 days in culture medium supplemented with recombinant IL2 and irradiated human serum (controlled source, male). On day 18, CAR-T cells were separated and residual TCRαβ^+^ cells removed. UCART19 cells were then cryopreserved and stored on day 19.

### UCART19 Cellular Kinetics by qPCR

Genomic DNA (gDNA) was isolated from peripheral blood (PB) and bone marrow (BM) aspirate samples by using a QIAamp DNA Blood Mini Kit (Qiagen, catalog no. 160015575). UCART19 transgene DNA was detected by using a TaqMan-based qPCR assay developed and validated by Navigate BioPharma Services. The number of copies/μg of DNA was quantified by using a 7-point standard curve containing 200 ng control gDNA spiked with 10 to 10^6^ copies of UCART19 plasmid DNA. A separate *P21/CDKN1A* qPCR assay run in parallel quantified the amount of the original input gDNA tested and provided a correction factor. The number of copies/μL of blood or BM was then calculated according to total DNA yield and specimen volume. The assay has a lower limit of quantification of 10 copies/μg of DNA requiring 200 ng DNA per reaction.

### UCART19 Cellular Kinetics and Immunophenotyping by Flow Cytometry

Fresh whole ethylenediaminetetraacetic acid (EDTA) blood and BM aspirate specimens were stained, acquired, and analyzed by multiparameter flow cytometry for detection of CAR^+^ T cells as detailed by Benjamin and colleagues (14). When possible, further characterization of the expanding CAR^+^-T cell subsets was performed, including CD4^+^:CD8^+^ ratio, and memory/effector CAR^+^-T cell subsets.

### Host Immune Cell Recovery by Flow Cytometry

The kinetics of host immune cell depletion and reconstitution [i.e.*,* percentages and absolute cell counts of T and natural killer (NK) subpopulations] resulting from lymphodepletion and/or UCART19 treatments was monitored by using a FACS Canto II Flow Cytometer (BD Biosciences) through a standard six-color flow cytometry panel including CD45, CD3, CD4, CD8, CD19, CD16/CD56 mAbs (BD Biosciences, catalog no. 644611, RRID:AB_2870318) combined with the BD Trucount technology (BD Biosciences, catalog no. 340334). Data analysis was performed using FACS Diva software (BD Biosciences).

### Alemtuzumab Pharmacokinetics

Four to 17 (median = 11) timepoints per patient were collected to determine the pharmacokinetics of alemtuzumab. Samples were spread between the end of the last infusion up to days 20 to 90 (depending on patient, median = 35 days). These profiles allowed determination of the AUC through a bicompartmental pharmacokinetic population-based model, performed on Monolix 2019R2 software. Sera were analyzed by an ELISA, using the Versamax ELISA-reader, Softmax Prio version 6 software, and anti-alemtuzumab antibodies produced by Geoff Hale Developments. The lower limit of quantification was 0.01 μg/mL.

### Monitoring of Soluble Immune Factors

Cryopreserved plasma samples collected every 1 to 4 days, from day-7 (prior to lymphodepletion) to day 28 after UCART19 infusion, were thawed and analyzed by sandwich electrochemiluminescence assay for measurement of IL2, IL4, IL6, IL7, IL10, IL15, TNFα, IFNγ, GMCSF, and C-reactive protein by using a MSD 96-Well V-PLEX Assay including several V-Plex panels (MSD, catalog no. K15049D, K15050D, and K151STD). Proteins of interest were captured by antibodies coated on a plate, then bound by a secondary detection antibody labeled with an MSD Sulfo-Tag. After adding the MSD read buffer and applying voltage, the intensity of emitted light was measured by the Meso QuickPlex SQ 120 imager and quantified according to calibration standards.

### Statistical Analysis

Classical noncompartmental analysis (NCA) parameters (*C*_max_, *T*_first_, *T*_max_, *T*_last_, AUC_14_, Auc_0-28_, AUCT_last_) were computed on various time-dependent variables, including UCART19, IL7, IL15, host T and NK cells. Below limit of quantification values for UCART19, host T and NK lymphocytes have been replaced by 0 and possible UCART19 observations during redistribution phases have been removed for NCA computation only. Statistical nonparametric (two-sided Wilcoxon rank-sum and Spearman) tests were then performed to explore relationships between these parameters and various patient or protocol covariates (R software, version 3.6.1).

### Data Availability

The data generated in this study are not publicly available as they include information that may compromise patient privacy. They may be available for scientific and medical professions upon reasonable request and following assessment of the request. Request should be sent to the corresponding author (sandra.dupouy@servier.com).

## Results

### Cellular Kinetics of UCART19 in PB

Data from 25 adult patients with R/R B-ALL who received UCART19 in the CALM study were included in the pharmacokinetic analysis. UCART19 transgene levels, evaluated by qPCR and represented as vector copy number (VCN), showed a positive correlation (rho = 0.85, *P* = 1.8e-06) between PB and BM aspirate (Supplementary Fig. S2). Results from PB only will be discussed in this article. Because of the variations induced by lymphodepleting chemotherapy on gDNA levels, UCART19 cellular kinetics has been expressed in copies/μL blood rather than copies/μg gDNA (28).

**FIGURE 1:**
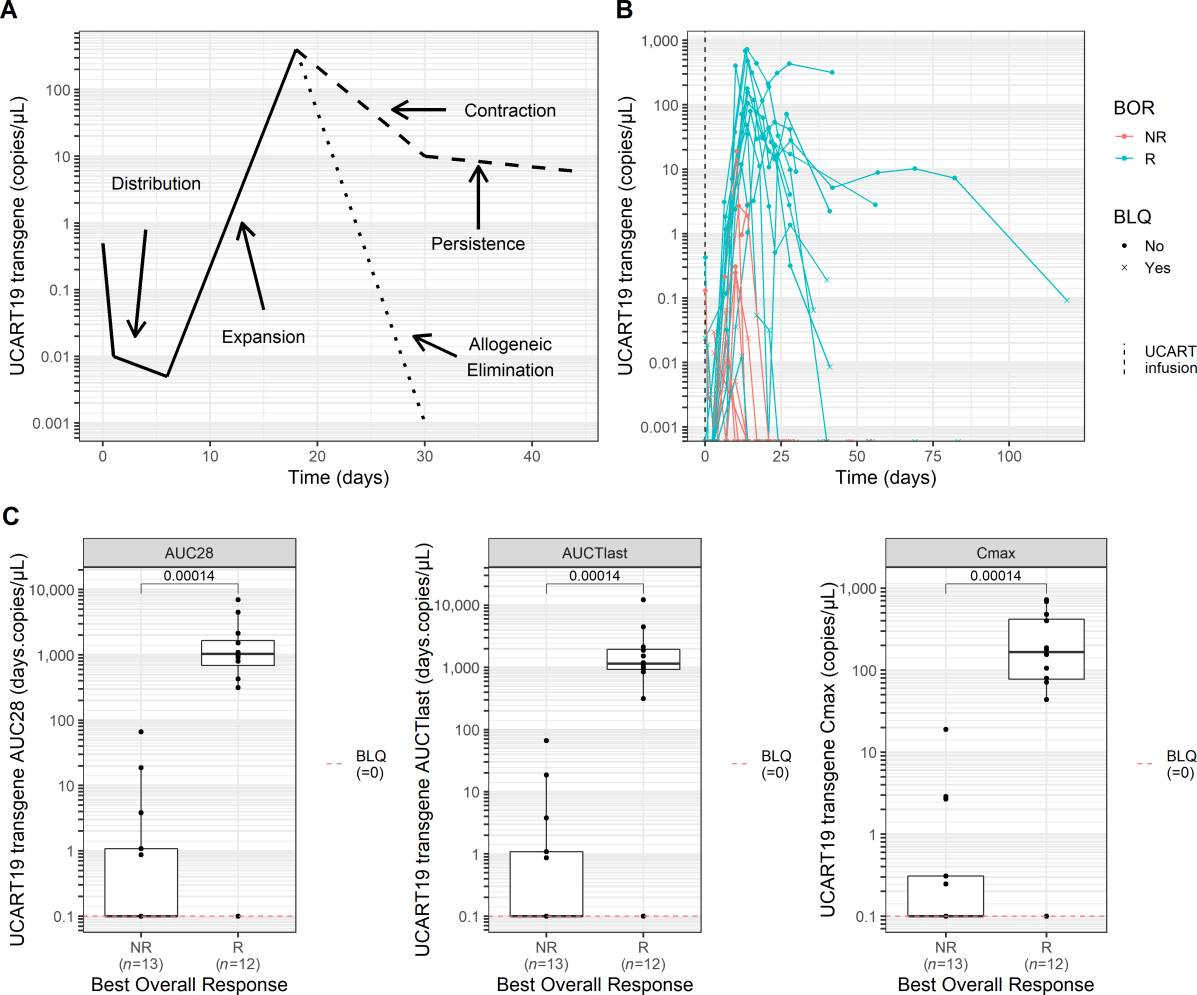
UCART19 cellular kinetics and correlation with clinical response. **A,** Graphical representation of UCART19 kinetic profile in PB of patients with B-ALL in the CALM study. **B,** Individual UCART19 kinetic profiles of adult patients with B-ALL by best overall response (BOR). **C,** From left to right: UCART19 AUC_0-28_ (AUC between day 0 and day 28 after CAR-T infusion), AUCT_last_ (AUC from day 0 until the last observed quantifiable level of CAR transgene) and *C*_max_ (maximum peak expansion) according to response status. Statistical comparison was performed using a Wilcoxon test. BLQ, below the limit of quantification; NR, nonresponder; R, responder.

A graphical representation of the UCART19 cellular kinetics profile is depicted in Fig. 1A. A few hours after infusion, circulating UCART19 levels dramatically decrease suggesting cell distribution throughout various tissues. Binding of CAR^+^ T cells to CD19^+^ target cells induces activation and exponential growth of UCART19 cells which is represented by a marked increase in the transgene levels starting from day 8 to day 14 after infusion. Once UCART19 reached maximum expansion in blood (*C*_max_), a rapid decline was observed in most patients by day 28, while in others, a biexponential decline occurred with a rapid initial contraction phase followed by a more stable and persistent phase.

Individual UCART19 cellular kinetic profiles and derived NCA parameters showed that peripheral CAR-T cell expansion was seen in 14 of 25 patients (56%), with a median time to maximal expansion (*T*_max_) of 14 days (range, 10–27 days) after first infusion. UCART19 cells then became undetectable with a median transgene persistence of 28 days (range, 10–82 days), except in 4 patients where low levels were observed in blood beyond 42 days, and up to 3 months in a single patient (Fig. 1B; Table 1). Because of the conditioning regimen used before subsequent allo-SCT, persistence was interrupted in all of these 4 patients (between day 47 and day 62). The median *C*_max_, exposure from day 0 to day 28 (AUC_0-28_), and until the last observed quantifiable level of transgene (AUCT_last_) showed a significantly higher expansion and persistence in the 12 (48%) responder patients compared with limited to no expansion in the 13 (52%) nonresponders (Fig. 1C).

**TABLE 1:** Summary of UCART19 cellular kinetic parameters in PB of responding and nonresponding adult patients with B-ALL (qPCR)

BOR	NR (*n* = 13)	R (*n* = 12)	Overall (*n* = 25)
***C*_max_ (copies/μL)**			
Mean (SD)	1.9 (5.21)	258.7 (250.28)	125.2 (214.17)
Median [min;max]	0 [0;18.9]	166.6 [0;721.7]	2.9 [0;721.7]
**AUC_0-28_ (days.copies/μL)**			
Mean (SD)	7 (18.57)	1725.4 (2021.32)	831.8 (1624.98)
Median [min;max]	0 [0;66.4]	1030.3 [0;6973.8]	18.6 [0;6973.8]
**AUCT_last_ (days.copies/μL)**			
Mean (SD)	7 (18.57)	2310.4 (3332.35)	1112.6 (2543.48)
Median [min;max]	0 [0;66.4]	1153.4 [0;12252.5]	18.6 [0;12252.5]
**Time**	**Expansion** **(*n* = 14)**		
***T*_first_ (days)**			
Mean (SD)	9 (2.79)		
Median [min;max]	8 [6.3;14]		
***T*_last_ (days)**			
Mean (SD)	31.6 (19.2)		
Median [min;max]	28 [10.4;82.1]		
***T*_max_ (days)**			
Mean (SD)	15.1 (4.65)		
Median [min;max]	14 [10;26.8]		

Abbreviations: AUC_0-28_, area under the curve between day 0 and day 28 after UCART19 infusion; AUCT_last_, area under the curve from day 0 until the last observed quantifiable level of CAR transgene; *C*_max_, maximum peak expansion; qPCR, quantitative polymerase chain reaction; *T*_first_, time of first quantifiable UCART19 transgene (samples were not collected at the same time points); *T*_last_, time of last quantifiable UCART19 transgene; *T*_max_, time of maximal expansion.

### UCART19 Dose and Cellular Kinetics

To establish the MTD of UCART19, the CALM trial included a dose-escalation phase. Patients received one of the three dose levels (DL) of total CAR^+^ T cells: 6 of 25 patients (24%) received 6 × 10⁶ cells (DL1), 6 (24%) received 6–8 × 10⁷ cells (DL2), and 7 (28%) received 1.8–2.4 × 10⁸ cells (DL3). A further 6 (24%) patients received DL2, determined as the recommended dose, during the expansion phase. Although the number of patients treated was limited, dose exposure analysis did not show any relationship between UCART19 DL and cellular kinetic parameters (*C*_max_, AUC_0-28_, AUCT_last_). Furthermore, in the 14 patients who experienced expansion, there was no difference in *T*_max_ between the three DLs (median = 13.1, 13.8, and 14 days for DL1, DL2, and DL3, respectively; Supplementary Table S2).

### Factors Associated with UCART19 Expansion and Persistence

The impact of various patient-related factors on cellular kinetic parameters (*C*_max_ and AUCT_last_) was assessed (Supplementary Figs. S3 and S4, respectively). Although no significant differences were reported with age, patient sex appeared to affect UCART19 expansion and persistence with a trend toward higher values in male compared with female patients (Supplementary Figs. S3A, S3B, S4A, and S4B). The fact that all UCART19 T-cell donors were male (Supplementary Table S3) suggests sex mismatch may have played a role.

We also evaluated the relationship between prior therapies and UCART19 expansion and persistence (Supplementary Figs. S3C, S3D, S4C, and S4D). Enrolled patients had received 1 to 6 (median = 4) previous lines of therapy including inotuzumab ozogamicin in 8 patients (32%) and blinatumomab in 12 patients (48%). Seven patients (28%) did not receive prior allo-SCT, 14 patients (56%) had received one, and 4 patients (16%) had received two. Our dataset analysis showed a negative correlation between the number of previous lines and UCART19 *C*_max_ (rho = −0.58, *P* = 0.0022) or AUCT_last_ (rho = −0.57, *P* = 0.0027). In addition, none of the 4 patients who received two prior allo-SCTs showed expansion and consequently, no persistence. All but one received FCA lymphodepletion prior to UCART19 administration.

The ratio of AUC_0-28_ to tumor burden has been described as a good indicator of the long-term prognosis of patients with B-ALL treated with autologous anti-CD19 CAR-T cells (16, 29). Our results showed that tumor burden at the time of UCART19 infusion did not seem to affect expansion and persistence of the transgene when pooling all the patients together (Supplementary Figs. S3E and S4E). However, a positive correlation was found between tumor burden and UCART19 AUCT_last_ and *C*_max_ (*P* < 0.01) when analyzing the responding patients (Supplementary Fig. S5), suggesting that tumor burden at the time of infusion may affect UCART19 pharmacokinetics in this subgroup.

### Impact of T-cell Donor and Product Characteristics on UCART19 Cellular Kinetics

Nine batches from 6 different donors were infused. Final products were characterized for CAR expression, CD52/TCRαβ knockout efficiency, memory markers, and CD4 or CD8 expression in CAR^+^ T cells (Supplementary Fig. S6). Mean levels of CAR^+^ T cells was 47.2%, SD = 10.4 (Supplementary Fig. S6A). Central memory (Tcm) and effector memory (Tem) T cells represented the major populations among αβ T cells (>70%; Supplementary Fig. S6B). Knockout efficiency in CAR^+^ T cells was also studied among batches (percentages of CD52^−^: mean = 68.5%, SD = 10.9; percentages of CD52^−^/TCRαβ^−^: mean = 65.3%, SD = 9.9; Supplementary Fig. S6C). An equivalent balance of CD4^+^ and CD8^+^/CAR^+^ cell lineages was found (Supplementary Fig. S6D). Residual TCRαβ expression and total CAR^+^/TCRαβ^−^ T cells (mean and SD) were also determined (Supplementary Fig. S6E). Mean fold-expansion of viable cells during the expansion phase of manufacturing, from day 6 until day 18 (mean = 14.4, SD = 9.7) did not show significant disparities among the different batches (Supplementary Fig. S6F). Although the manufacturing process lasted 19 days, we considered that the expansion phase only started after the TALEN® electroporation step (day 6) and ended before the TCRαβ^+^ purification step (day 18), to avoid introducing any biases inherent to these two steps.

We next investigated the relationship between the features of the final product (drug substance) and *in vivo* UCART19 cellular kinetics.

**FIGURE 2:**
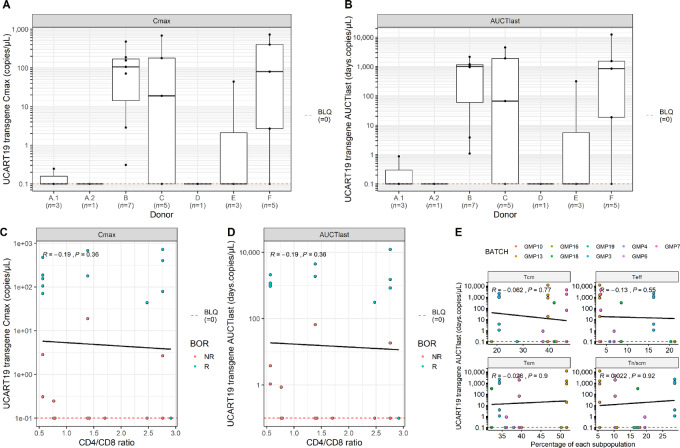
Impact of T-cell donor and product characteristics on UCART19 cellular kinetics. Peak (*C*_max_; **A**) and persistence (AUCT_last_; **B**) of UCART19 expansion related to donors are represented. Graphs show mean ± SD values of transgene levels by VCN analysis (copies/μL). Number of patients having received a batch from the same donor (*n*) are shown. Correlation between peak (**C**) and persistence (**D**) of UCART19 expansion related to the CD4:CD8 ratio of CAR^+^ cells in the drug substance (DS) is analyzed. DS characterization is performed by multiparametric flow cytometry at D19 of manufacturing. **E,** Persistence of UCART19 (AUCT_last_) depending on memory subsets composition of the GMP batches is evaluated. Statistical comparison was performed using a Wilcoxon test for **A** and **B** and a Spearman correlation for **C**–**E**. [Tn, naïve T cells and Tscm, stem cell memory T cells (CD62L^+^ CD45RA^+^); Tcm, central memory T cells (CD62L^+^ CD45RA^−^); Tem, effector memory T cells (CD62L^−^ CD45RA^−^); Teff, effector T cells (CD62L^−^ CD45RA^+^)].

Characteristics of healthy T-cell donors (age, sex, body mass index, ABO, and Rh) are presented in Supplementary Table S3. The impact of each donor on UCART19 *C*_max_ and AUCT_last_ was further assessed (Fig. 2A and B). However, due to the low number of patients treated with a single batch, further data including a deeper analysis of T-cell fitness are required to conclude on the relevance of donor impact.

The CD4 and CD8 composition was previously shown to be of relevance in some autologous anti-CD19 CAR-T cell studies (22, 26, 30). Therefore, we evaluated the impact of the CD4^+^:CD8^+^ ratio on UCART19 *in vivo* expansion and persistence (Fig. 2C and D). No significant correlation was observed (*P* = 0.36 for both *C*_max_ and AUCT_last_).

Enrichment of early memory T-cell subpopulations in the infused product is also known to positively affect clinical outcome in B-cell malignancies (22, 27). No relationship between specific enrichment in a memory subtype and *in vivo* persistence (AUCT_last_) of UCART19 was shown [*P* = 0.77 and 0.92 for Tcm and naïve/stem cell memory T cells (Tn/Tscm), respectively] (Fig. 2E). In addition, *in vivo* UCART19 expansion and persistence were also evaluated with regards to transduction efficiency and viability of the infused batches, without any significant correlation (Supplementary Fig. S7). Overall, based on the parameters investigated, we were not able to show any clear impact of product characteristics on UCART19 kinetics. Additional data are required to allow a deeper knowledge of the impact of product attributes on UCART19 expansion and persistence.

### Impact of Lymphodepletion and Alemtuzumab on UCART19 Cellular Kinetics

**FIGURE 3:**
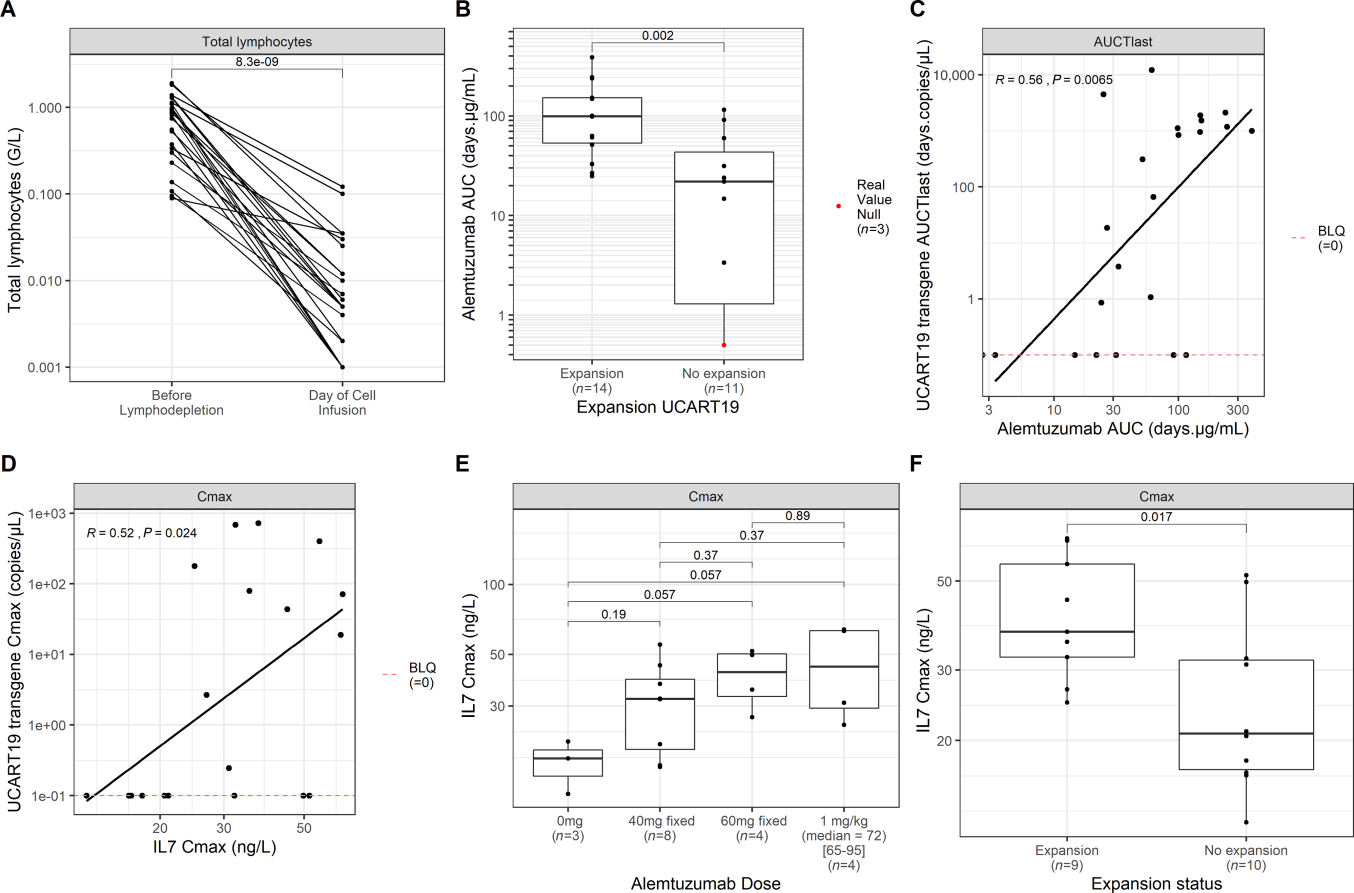
Impact of lymphodepletion and alemtuzumab on UCART19 cellular kinetics. **A,** Total lymphocytes before and after (at the day of UCART19 infusion) lymphodepletion. **B,** Relationship between alemtuzumab total exposure and UCART19 expansion status. **C,** Positive correlation between alemtuzumab and UCART19 exposure. **D,** Correlation between UCART19 and IL7 *C*_max_. **E,** Relationship between IL7 *C*_max_ and alemtuzumab doses. **F,** Relationship between IL7 *C*_max_ and UCART19 expansion status. Statistical comparison was performed using a Wilcoxon test for **B**, **E**, and **F** and a Spearman correlation for **D**.

Prior to UCART19 infusion, all patients received a lymphodepletion regimen comprising fludarabine (F, 90 mg/m^2^) and cyclophosphamide (C, 1,500 mg/m^2^) with or without alemtuzumab (A, 1 mg/kg, or 40 mg, or 60 mg flat dose), to improve CAR-T cell engraftment and expansion. The impact of these regimens on total host lymphocyte count is shown in Fig. 3A. We have previously reported that alemtuzumab-containing lymphodepletion appears to be required for UCART19 expansion (14, 15). To confirm these results, we explored the relationship between alemtuzumab doses and UCART19 expansion across the three DLs of UCART19 (Supplementary Table S4). Three of the 25 patients received an FC regimen only and none of them experienced UCART19 expansion. The other 22 patients received five alemtuzumab administrations in addition to the FC regimen (FCA). Alemtuzumab total flat doses of 40 and 60 mg led to 44% (4/9) and 50% (2/4) expansion rates, respectively, while the weighted dose of 1 mg/kg (median = 72 mg, range 65–95) resulted in 88% (8/9) expansion rate. In addition to alemtuzumab dose, we also assessed the impact of its exposure (AUC) on UCART19 expansion and persistence. Figure 3B shows that patients who experienced UCART19 expansion had a significantly greater alemtuzumab AUC compared with those who did not have expansion (median = 99 vs. 21 days.μg/mL; *P* = 0.002). A significant positive correlation (rho = 0.56, *P* = 0.0065) was also found between alemtuzumab exposure and UCART19 AUCT_last_ (Fig. 3C).

It was previously reported that lymphodepletion drives higher levels of homeostatic cytokines, such as IL7 and IL15, which enhances autologous CAR-T cell expansion (19, 20, 31). We investigated the impact of FC and FCA regimens on IL7 and IL15 circulating levels (Supplementary Fig. S8). Both cytokines were effectively increased after lymphodepletion (Supplementary Fig. S8A). Unexpectedly, neither IL15 *C*_max_ nor exposure from first measurement until the first 2 weeks following UCART19 infusion (AUC_14_) was significantly correlated with UCART19 expansion (Supplementary Fig. S8B, S8C, and S8E). However, IL7 *C*_max_ was positively correlated with UCART19 kinetics (rho = 0.52, *P* = 0.024), with significantly higher IL7 *C*_max_ (median = 41 vs. 21 ng/L, *P* = 0.017) and AUC_14_ (median = 484 vs. 293 day.ng/L, *P* = 0.043) in patients with CAR expansion (Fig. 3D–F; Supplementary Fig. S8D). The levels of other tested cytokines were not found to correlate with UCART19 kinetics.

Given the relationship between alemtuzumab dose and UCART19 expansion, and the correlation between IL7 and UCART19 kinetics, we assessed the impact of alemtuzumab dose on IL7 *C*_max_. Figure 3E shows a dose-dependent relationship between both parameters, with an IL7 level plateau reached at doses equal or higher to 60 mg of alemtuzumab.

### Impact of Alemtuzumab on Host T-cell and NK-cell Kinetics

**FIGURE 4:**
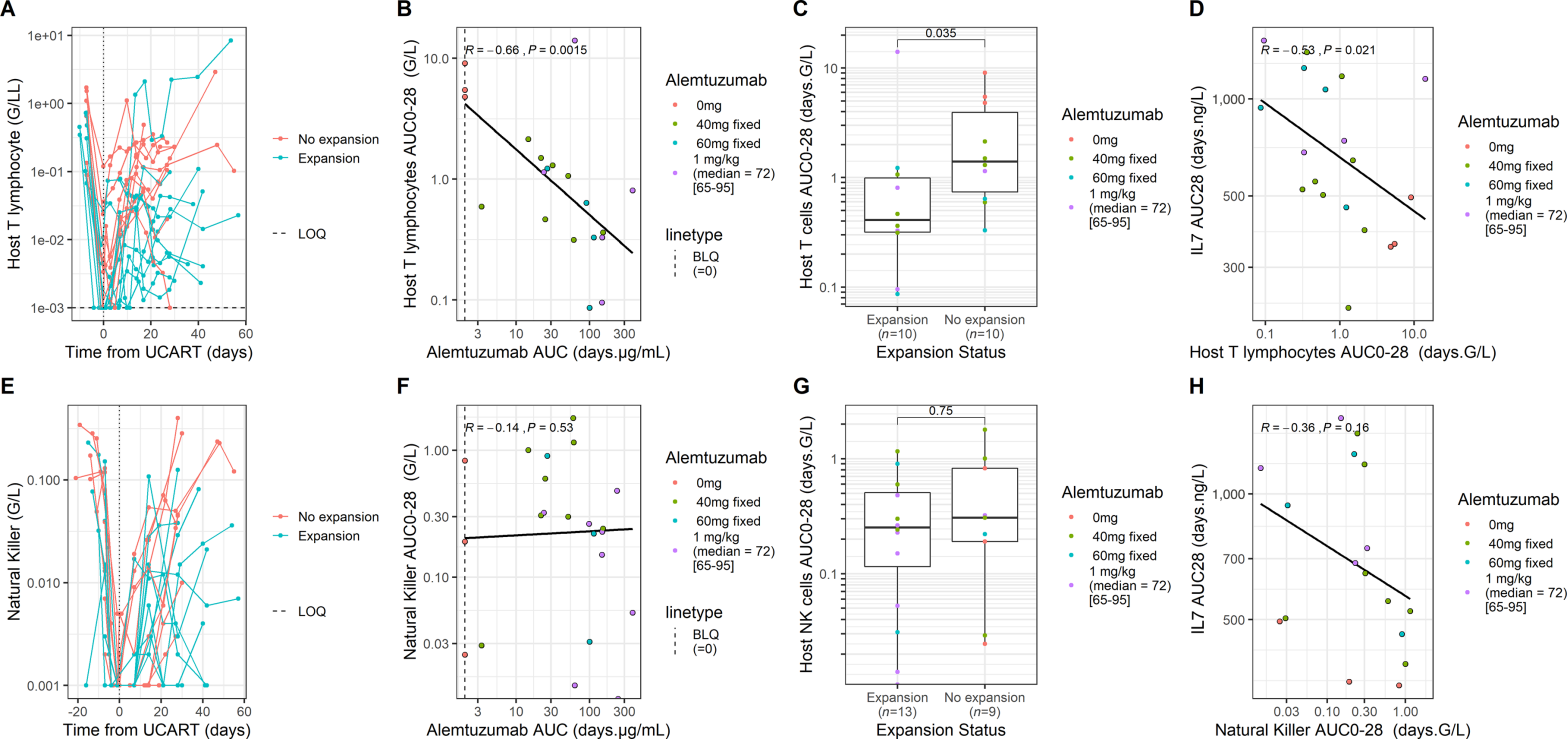
Interplay between alemtuzumab, IL7, host immune system and UCART19 kinetics. Individual host T profiles up to day 60, highlighted by expansion status (**A**), relationship between alemtuzumab total exposure and host T early AUC (AUC_0-28_; **B**), higher host T early exposures are correlated with the absence of observable UCART19 expansion (**C**), IL7 exposure AUC_28_ and host T early exposure AUC_0-28_ are strongly correlated (**D**). Individual host NK profiles up to day 60, highlighted by expansion status (**E**), relationship between alemtuzumab total exposure and host NK early AUC (AUC_0-28_; **F**), absence of correlation between host NK early exposures and UCART19 expansion (**G**), absence of correlation between IL7 exposure AUC_28_ and host NK early exposure AUC_0-28_ (**H**). Statistical comparison was performed using a Wilcoxon test for **C** and **G** and a Spearman correlation for **B**, **D**, **F**, and **H**.

Given the crucial role of alemtuzumab on UCART19 kinetics, its impact on host cell populations that may be involved in early allogeneic cell–mediated rejection was explored (Fig. 4).

The individual host peripheral T- and NK-cell profiles measured by flow cytometry are shown up to day 56 in Fig. 4A–E, respectively. We further investigated the impact of alemtuzumab dose and exposure on both host T- and NK-cell exposure within the first 28 days after UCART19 administration. A negative correlation between host T lymphocyte AUC_0-28_ and both alemtuzumab exposure (rho = −0.66, *P* = 0.0015) and UCART19 expansion (median = 0.41 vs. 1.40, *P* = 0.035) was observed (Fig. 4B and C). However, we did not observe a clear correlation between NK cell AUC_0-28_ and alemtuzumab exposure (*P* = 0.53) or UCART19 expansion status (*P* = 0.75; Fig. 4F and G).

Figure 3 shows a significant correlation between alemtuzumab exposure, IL7 levels, and UCART19 expansion. Interestingly, unlike NK cells, a significant negative correlation exists between IL7 exposure [computed from first measurement until 28 days following UCART19 infusion (AUC_28_)] and host T lymphocyte exposure AUC_0-28_ (rho = −0.53, *P* = 0.021; Fig. 4D–H).

### Impact of HLA Allele Matching Disparities Between Donors and Recipients and UCART19 Kinetics

**FIGURE 5:**
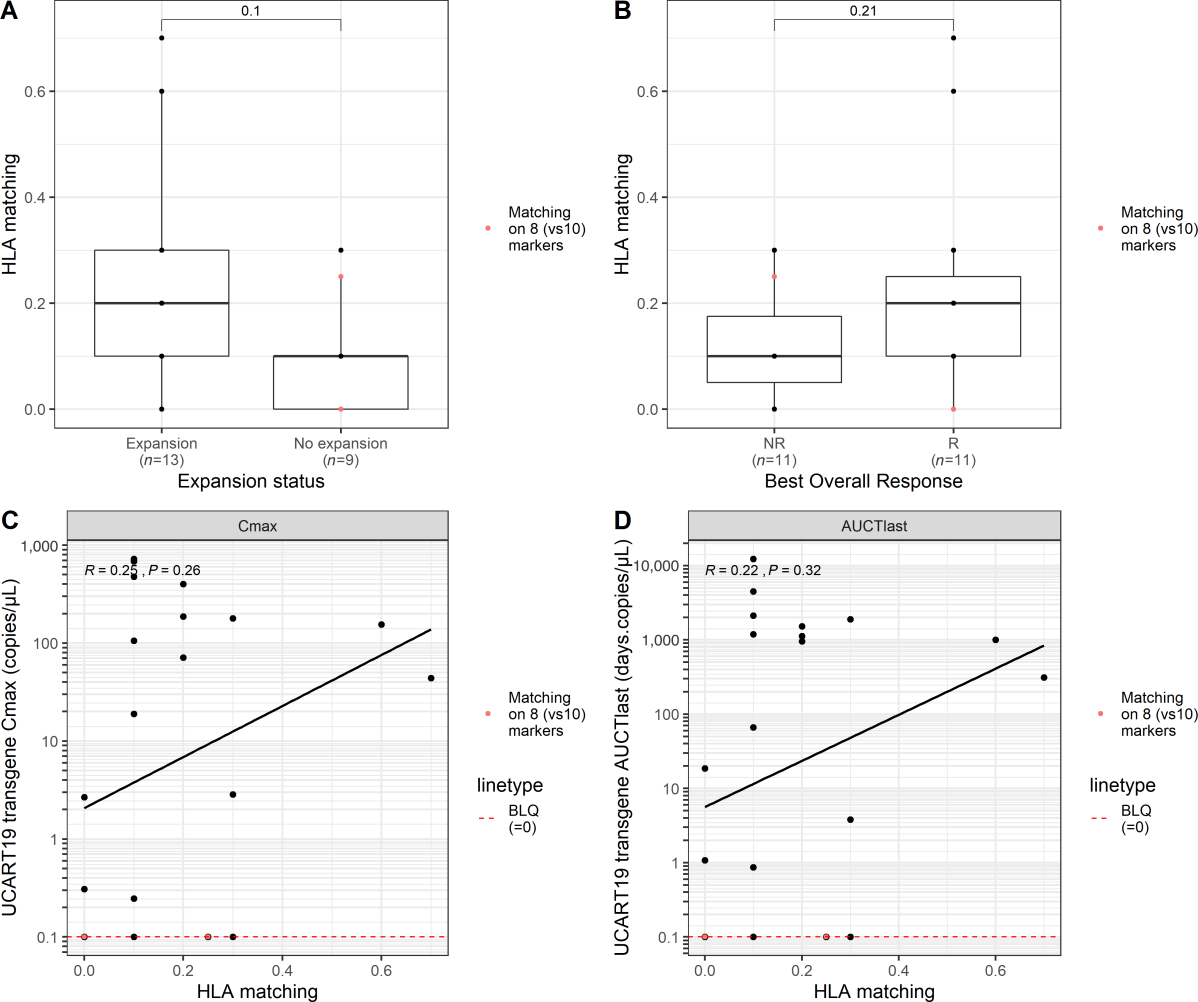
Influence of HLA allele mismatching between unrelated UCART19 T-cell donors and recipients on UCART19 kinetics. Level of HLA matching (expressed as a percentage of overall matching) represented according to the UCART19 expansion status (**A**) or, clinical response status (**B**). Comparison is based on eight alleles (red dots) or 10 alleles (black dots) depending on local practices for HLA allele typing assessment. Relationship between HLA matching level and UCART19 *C*_max_ (**C**) or, AUCT_last_ (**D**). Statistical comparison was performed using a Wilcoxon test for **A** and **B** and a Spearman correlation for **C** and **D**.

The degree of donor-recipient HLA allele matching between UCART19 T-cell donor and patient was evaluated to assess its impact on UCART19 kinetics and response. On the basis of a compatibility analysis on eight or 10 alleles (depending on standard practices in the different countries involved in the CALM study), more than 90% of patients (20/22) for whom HLA allele typing comparison was possible displayed less than 30% HLA matches, the remaining 9% patients (2/22) showed more than 60% matching. Three patients were not assessable due to the use of nonequivalent methodology. The degree of donor-recipient HLA matching was correlated with the status of UCART19 expansion (Fig. 5A) and response (Fig. 5B) but was not significant with this limited number of patients (*P* = 0.1 and *P* = 0.21, respectively). The 2 patients with the highest HLA allele matching (i.e., ≥ 60%) were responders, both reaching molecular remission at day 28 but with a variable duration of response. On the other hand, some responder patients showed no to very low matching (i.e. <30%). When considering the cellular kinetic parameters, we did not demonstrate any correlation between the level of HLA matching and UCART19 *C*_max_ (*P* = 0.26) or AUCT_last_ (*P* = 0.32; Fig. 5C and D). Among the patients with the highest UCART19 peak expansion or the longest persistence, the level of HLA matching was less than 30%, indicating that other parameters may have more impact on UCART19 kinetics and response in this allogeneic setting.

## Discussion

CAR-T cell therapies are complex products with unique pharmacologic properties. Most of our current knowledge on their pharmacokinetics and pharmacodynamics is gathered from the clinical trials of patient-derived autologous anti-CD19 CAR-T cells in B-cell malignancies. However, given the increased interest in allogeneic “off-the-shelf" CAR-T cell products, several questions regarding their safety, efficacy, and clinical pharmacology still need to be answered to efficiently support their clinical development.

We previously showed that UCART19, an allogeneic genome-edited anti-CD19 CAR-T cell therapy, could be safely administered and achieved antileukemic activity in pediatric and adult patients with R/R B-ALL enrolled in two multicenter phase I studies (14, 15).

Here, we present the first comprehensive report on UCART19 cellular kinetics in adult patients with R/R B-ALL, who underwent conditioning with fludarabine/cyclophosphamide ± alemtuzumab in the CALM trial. Although a small number of patients was included in this analysis, these results provide several highlights that could be further explored for better understanding of allogeneic CAR-T cell kinetics and determinants of response.

The clinical efficacy of autologous CAR-T cells in patients with B-ALL was shown to positively correlate with higher peak expansion, exposure, and persistence in responder patients as compared with nonresponders (1, 2, 16, 17, 21, 32–34). A similar correlation was observed with UCART19 as both *C*_max_ and AUC_0-28_ significantly correlated with clinical response, confirming the importance of understanding the driving factors of UCART19 pharmacokinetics. To evaluate the interplay between each previously reported parameter (patient demographics, previous treatments, tumor burden, product characteristics, and lymphodepletion) and UCART19 kinetics, we performed univariate analyses.

Our results show that UCART19 shares similar early cellular kinetics with autologous second generation anti-CD19 CAR-T cell therapies (1, 16, 21, 32, 33). Generally, UCART19 cells undergo a rapid decline after infusion, probably representing an initial distribution phase into several tissues. Once CAR^+^ T cells encounter CD19^+^ cells, they expand in PB, and reach a peak of proliferation (*C*_max_) within 2 weeks after infusion. It is important to notice that engraftment varies between patients, with highest *C*_max_ values that are quite similar to those reported by Mueller and colleagues and others in autologous setting (∼1e+05 copies/μg DNA; refs. 33, 34).

Also, as observed with autologous CAR-T cell therapies (30, 33), no correlation was found between UCART19 administered cell dose and UCART19 expansion or overall exposure, as measured by *C*_max_ and mean AUC from day 0 to day 28 (AUC_0-28_).

With regards to persistence, the CAR transgene concentration tends to decline rapidly in most patients, thus limiting the median persistence of UCART19 to 28 days. Nevertheless, 4 patients in the study had quantifiable UCART19 levels beyond day 42. Analysis of UCART19 cellular kinetics in these patients showed a biphasic elimination, with a rapid contraction phase followed by a slower decline that occurred over several weeks. One patient showed quantifiable UCART19 levels up to 3 months following infusion. Of note, all of them received additional conditioning prior to subsequent allo-SCT while still having measurable levels of UCART19. This conditioning ablated residual UCART19 and artificially reduced its persistence.

Three patients were conditioned with fludarabine and cyclophosphamide only. Interestingly, none of them displayed CAR-T cell peak, suggesting that, differently from autologous setting, increasing intensity of the lymphodepletion regimen with an anti-CD52 antibody (or through another strategy), may be required for the expansion of allogeneic CAR-T cells.

Several hypotheses, likely in combination, may explain the absence of expansion and/or acute contraction of UCART19 in some patients. First, CAR-T cell engraftment was shown to be influenced by lymphodepletion of the patient prior to product infusion (35). Turtle and colleagues showed that adding fludarabine to cyclophosphamide was superior to cyclophosphamide-only lymphodepletion regimens, both in terms of CAR-T cell expansion, persistence, and clinical outcomes (34). Several hypotheses have been suggested to explain the need for lymphodepletion such as its role in the elimination of immunosuppressive elements (e.g., regulatory T cells, myeloid-derived suppressor cells, and indoleamine 2,3-dioxygenase; refs. 35–37). The risk of host immune cells targeting CAR-T cells is theoretically even more prevailing with allogeneic products such as UCART19. This may explain why none of our patients without alemtuzumab had an expansion (and thus a clinical response), while alemtuzumab is generally not used in autologous CAR-T cell clinical trials.

It has also been reported that cell-mediated allogeneic rejection can be caused by residual host immunity or early host immune cell reconstitution in the context of an HLA-mismatched setting (11). In accordance, we found a negative relationship between host T-cell exposure (AUC_0-28_) and UCART19 expansion status. It supports the hypothesis that host T lymphocytes may have played a role in mediating elimination of UCART19. No similar correlation was seen with host NK cells in this study.

The second role of lymphodepletion is the elimination of homeostatic cytokine sinks inducing a favorable cytokine profile (such as IL7, IL15) for CAR-T cell activation and proliferation (19, 20, 31, 38). In physiologic conditions, IL7 and IL15 control proliferation and survival of the Tn and Tscm subpopulations, respectively. In the case of lymphodepletion, they act in synergy to facilitate lymphocyte repletion (39). Hirayama and colleagues (20) showed that in patients with aggressive non–Hodgkin lymphoma, a better progression-free survival was correlated with cytokines such as IL7 but not with lymphodepletion intensity. This is in accordance with our findings, as IL7 levels were well correlated with UCART19 expansion. On the other hand, IL15 was not correlated with UCART19 kinetics, as previously reported by some autologous CAR-T cell studies (31). One hypothesis could be that IL15 had reached levels provoking maximal effect in all UCART19-treated patients.

In addition, the number of prior lines of treatment was also reported to potentially reduce expansion of autologous CAR-T cells due to impairment of patient Tn and Tscm subpopulations and fitness (40). Although such an effect is not expected with CAR-T cells derived from healthy donors, UCART19 kinetics is also negatively affected by the number of prior lines, which may be related to a more aggressive disease phenotype or altered cytokine environment due to repeated treatments. Interestingly, the number of prior lines was not correlated with IL7 baseline level (prior to lymphodepletion) but was negatively correlated with IL7 AUC_0-28_ (Supplementary Fig. S9).

Finally, sex mismatch between UCART19 PBMC donors, who were all males, and patient recipients tended to affect UCART19 cellular kinetics parameters with lower *C*_max_ and AUCT_last_ in female (F) compared to male (M) patients. In HLA-matched unrelated allo-SCT, sex mismatch is a known deleterious factor resulting in graft-versus-host or host-versus-graft reactions (41–43). The role of minor histocompatibility antigens expressed on donor cells, such as H-Y antigens encoded by the Y chromosome, has been highlighted (44). These H-Y antigens can be recognized by female T cells resulting either in GVHD in F→M transplantation or in graft rejection in M→F transplantation. In addition, females may also have developed alloimmunity against these H-Y antigens during previous pregnancies with male fetuses. The observation of a negative correlation between sex mismatch and UCART19 expansion kinetics needs further exploration in future allogeneic CAR-T cell clinical studies.

Unlike previous mentioned factors, some others did not show any correlation with UCART19 kinetics, or available data were insufficient to enable firm conclusions to be drawn. Tumor burden at the time of UCART19 infusion did not significantly correlate with UCART19 cellular kinetics in contrast to previous autologous CAR-T cell studies that have shown a positive correlation between CAR-T cell expansion and overall tumor burden or CD19^+^ cells in BM (16, 26, 29, 33, 45). However, our analysis showed that in responding patients, tumor burden may affect the cellular kinetics of UCART19. At a tumor burden greater than 75%, no responder patients were observed, which could be explained by other intrinsic factors, such as activation-induced cell death (AICD), tonic signaling, and exhaustion, all of which have been reported to limit persistence of CAR-T cells (46–49). While tonic signaling and AICD markers were not evaluated in this study, T-cell exhaustion markers such as PD1, TIM3, and LAG3 were assessed by flow cytometry, but the limited data obtained did not enable a conclusion to be made. Finally, CAR-T cell kinetics was previously shown to be affected by the qualitative and quantitative characteristics of the infused product, such as enrichment in early memory subpopulations, CD4^+^:CD8^+^ ratio, and polyfunctionality of T cells (26, 27, 34, 50). Except for polyfunctionality which was not investigated in this study, none of the product characteristics analyzed were correlated with UCART19 expansion, nor was persistence. However, due to the low number of patients treated with the same UCART19 batch, further investigations including more parameters, such as CAR potency and T-cell fitness, are needed to increase our understanding of the impact of healthy donor–derived T cells on CAR-T cell kinetics.

Through gathering translational data of the CALM study, we hypothesize that intensive lymphodepletion strategies which include alemtuzumab, decrease host T lymphocytes, which may reject CAR-T cells in the allogeneic context, and increase availability of the homeostatic cytokine IL7 for CAR-T cell expansion. Controlling the interplay between these factors may facilitate better engraftment and persistence of UCART19 and consequently a better clinical outcome for patients. This interplay has been further investigated in a companion paper through a pharmacokinetic/pharmacodynamic model, simultaneously modeling total lymphocytes (with the integration of host T cells), IL7, and UCART19 (51). Of note, the model was not able to precisely dissociate the relative impact of IL7 and host T lymphocytes on UCART19 kinetics due to their correlation and the small number of patients in the study.

In conclusion, UCART19 was shown to proliferate and induce responses in adult patients with B-ALL following a lymphodepleting regimen including fludarabine, cyclophosphamide, and alemtuzumab. Several factors potentially influencing UCART19 cellular kinetics were identified, highlighting areas for improvement. Further efforts are needed to optimize the therapeutic window allowing appropriate expansion and persistence of allogeneic CAR-T cells among which, optimization of the chosen lymphodepletion regimen and strategy of redosing are key to making allogeneic CAR-T cell therapy a success.

## Supplementary Material

Supplementary Materials & Methods SM1 Additional information regarding the CALM study (including study population and summary results) and UCART19 product characterization.

Supplementary Figures S1-S9 Fig.S1: Schematic diagram of CALM study design; Fig.S2: Correlation of UCART19 transgene levels evaluated by qPCR in paired peripheral blood and bone marrow aspirate samples; Fig.S3 & S4: Impact of demographic characteristics, prior therapies and tumor burden on UCART19 in vivo expansion (Cmax) or persistence (AUCTlast), respectively; Fig.S5: Impact of tumor burden at the time of UCART19 infusion on UCART19 kinetics based on response status; Fig.S6: UCART19 product characteristics; Fig.S7: Scatter plots of UCART19 cellular kinetic parameters by qPCR vs transduction efficiency and cell viability; Fig.S8: Impact of lymphodepletion on homeostatic cytokines (IL-7 and IL-15) and UCART19 cellular kinetics; Fig.S9: Impact of number of prior treatment lines on IL-7 exposure (AUC28).

Supplementary Tables S1-S4 Table S1: Representativeness of CALM study participants; Table S2: UCART19 dose and cellular kinetics analysis; Table S3: Characteristics of UCART19 PBMC donors; Table S4: Impact of UCART19 and alemtuzumab doses on UCART19 expansion.

## References

[1] Maude SL , Laetsch TW, Buechner J, Rives S, Boyer M, Bittencourt H, . Tisagenlecleucel in children and young adults with B-cell lymphoblastic leukemia. N Engl J Med 2018;378:439–48. 29385370 10.1056/NEJMoa1709866 PMC5996391

[2] Neelapu SS , Locke FL, Bartlett NL, Lekakis LJ, Miklos DB, Jacobson CA, . Axicabtagene ciloleucel CAR T-cell therapy in refractory large B-cell lymphoma. N Engl J Med 2017;377:2531–44. 29226797 10.1056/NEJMoa1707447 PMC5882485

[3] Wang M , Munoz J, Goy A, Locke FL, Jacobson CA, Hill BT, . KTE-X19 CAR T-cell therapy in relapsed or refractory mantle-cell lymphoma. N Engl J Med 2020;382:1331–42. 32242358 10.1056/NEJMoa1914347 PMC7731441

[4] Abramson JS , Palomba ML, Gordon LI, Lunning MA, Wang M, Arnason J, . Lisocabtagene maraleucel for patients with relapsed or refractory large B-cell lymphomas (TRANSCEND NHL 001): a multicentre seamless design study. Lancet 2020;396:839–52. 32888407 10.1016/S0140-6736(20)31366-0

[5] Moreno-Cortes E , Forero-Forero JV, Lengerke-Diaz PA, Castro JE. Chimeric antigen receptor T cell therapy in oncology - pipeline at a glance: analysis of the ClinicalTrials.gov database. Crit Rev Oncol Hematol 2021;159:103239. 33497760 10.1016/j.critrevonc.2021.103239

[6] Shah BD , Ghobadi A, Oluwole OO, Logan AC, Boissel N, Cassaday RD, . KTE-X19 for relapsed or refractory adult B-cell acute lymphoblastic leukaemia: phase 2 results of the single-arm, open-label, multicentre ZUMA-3 study. Lancet North Am Ed 2021;398:491–502. 10.1016/S0140-6736(21)01222-8 PMC11613962 34097852

[7] Ying Z , Yang H, Guo Y, Li W, Zou D, Zhou D, . Relmacabtagene autoleucel (relma-cel) CD19 CAR-T therapy for adults with heavily pretreated relapsed/refractory large B-cell lymphoma in China. Cancer Med 2021;10:999–1011. 33382529 10.1002/cam4.3686 PMC7897944

[8] Fowler NH , Dickinson M, Dreyling M, Martinez-Lopez J, Kolstad A, Butler J, . Tisagenlecleucel in adult relapsed or refractory follicular lymphoma: the phase 2 ELARA trial. Nat Med 2022;28:325–32. 34921238 10.1038/s41591-021-01622-0

[9] Salmikangas P , Kinsella N, Chamberlain P. Chimeric antigen receptor T-cells (CAR T-cells) for cancer immunotherapy—moving target for industry? Pharm Res 2018;35:152. 29855723 10.1007/s11095-018-2436-z PMC5982434

[10] Lin JK , Muffly LS, Spinner MA, Barnes JI, Owens DK, Goldhaber-Fiebert JD. Cost effectiveness of chimeric antigen receptor T-cell therapy in multiply relapsed or refractory adult large B-cell lymphoma. J Clin Oncol 2019;37:2105–19. 31157579 10.1200/JCO.18.02079

[11] Depil S , Duchateau P, Grupp SA, Mufti G, Poirot L. 'Off-the-shelf' allogeneic CAR T cells: development and challenges. Nat Rev Drug Discov 2020;19:185–99. 31900462 10.1038/s41573-019-0051-2

[12] Philip B , Kokalaki E, Mekkaoui L, Thomas S, Straathof K, Flutter B, . A highly compact epitope-based marker/suicide gene for easier and safer T-cell therapy. Blood 2014;124:1277–87. 24970931 10.1182/blood-2014-01-545020

[13] Poirot L , Philip B, Schiffer-Mannioui C, Le Clerre D, Chion-Sotinel I, Derniame S, . Multiplex genome-edited T-cell manufacturing platform for "Off-the-Shelf" adoptive T-cell immunotherapies. Cancer Res 2015;75:3853–64. 26183927 10.1158/0008-5472.CAN-14-3321

[14] Benjamin R , Graham C, Yallop D, Jozwik A, Mirci-Danicar OC, Lucchini G, . Genome-edited, donor-derived allogeneic anti-CD19 chimeric antigen receptor T cells in paediatric and adult B-cell acute lymphoblastic leukaemia: results of two phase 1 studies. Lancet 2020;396:1885–94. 33308471 10.1016/S0140-6736(20)32334-5 PMC11773457

[15] Benjamin R , Jain N, Maus MV, Boissel N, Graham C, Jozwik A, . UCART19, a first-in-class allogeneic anti-CD19 chimeric antigen receptor T-cell therapy for adults with relapsed or refractory B-cell acute lymphoblastic leukaemia (CALM): a phase 1, dose-escalation trial. Lancet Haematol 2022;9:e833–43. 36228643 10.1016/S2352-3026(22)00245-9 PMC11575699

[16] Mueller KT , Maude SL, Porter DL, Frey N, Wood P, Han X, . Cellular kinetics of CTL019 in relapsed/refractory B-cell acute lymphoblastic leukemia and chronic lymphocytic leukemia. Blood 2017;130:2317–25. 28935694 10.1182/blood-2017-06-786129 PMC5731220

[17] Milone MC , Bhoj VG. The pharmacology of T cell therapies. Mol Ther Methods Clin Dev 2018;8:210–21. 29552577 10.1016/j.omtm.2018.01.010 PMC5852291

[18] Park JH , Geyer MB, Brentjens RJ. CD19-targeted CAR T-cell therapeutics for hematologic malignancies: interpreting clinical outcomes to date. Blood 2016;127:3312–20. 27207800 10.1182/blood-2016-02-629063 PMC4929923

[19] Neelapu SS . CAR-T efficacy: is conditioning the key? Blood 2019;133:1799–800. 31023743 10.1182/blood-2019-03-900928

[20] Hirayama AV , Gauthier J, Hay KA, Voutsinas JM, Wu Q, Gooley T, . The response to lymphodepletion impacts PFS in patients with aggressive non-Hodgkin lymphoma treated with CD19 CAR T cells. Blood 2019;133:1876–87. 30782611 10.1182/blood-2018-11-887067 PMC6484391

[21] Park JH , Rivière I, Gonen M, Wang X, Sénéchal B, Curran KJ, . Long-term follow-up of CD19 CAR therapy in acute lymphoblastic leukemia. N Engl J Med 2018;378:449–59. 29385376 10.1056/NEJMoa1709919 PMC6637939

[22] Locke FL , Rossi JM, Neelapu SS, Jacobson CA, Miklos DB, Ghobadi A, . Tumor burden, inflammation, and product attributes determine outcomes of axicabtagene ciloleucel in large B-cell lymphoma. Blood Adv 2020;4:4898–911. 33035333 10.1182/bloodadvances.2020002394 PMC7556133

[23] Cheadle EJ , Hawkins RE, Batha H, O'Neill AL, Dovedi SJ, Gilham DE. Natural expression of the CD19 antigen impacts the long-term engraftment but not antitumor activity of CD19-specific engineered T cells. J Immunol 2010;184:1885–96. 20089697 10.4049/jimmunol.0901440

[24] Weinkove R , George P, Dasyam N, McLellan AD. Selecting costimulatory domains for chimeric antigen receptors: functional and clinical considerations. Clin Transl Immunology 2019;8:e1049. 31110702 10.1002/cti2.1049 PMC6511336

[25] Kawalekar OU , O'Connor RS, Fraietta JA, Guo L, McGettigan SE, Posey AD, . Distinct signaling of coreceptors regulates specific metabolism pathways and impacts memory development in CAR T cells. Immunity 2016;44:380–90. 26885860 10.1016/j.immuni.2016.01.021 PMC13498396

[26] Turtle CJ , Hanafi LA, Berger C, Gooley TA, Cherian S, Hudecek M, . CD19 CAR-T cells of defined CD4+:CD8+ composition in adult B cell ALL patients. J Clin Invest 2016;126:2123–38. 27111235 10.1172/JCI85309 PMC4887159

[27] Fraietta JA , Lacey SF, Orlando EJ, Pruteanu-Malinici I, Gohil M, Lundh S, . Determinants of response and resistance to CD19 chimeric antigen receptor (CAR) T cell therapy of chronic lymphocytic leukemia. Nat Med 2018;24:563–71. 29713085 10.1038/s41591-018-0010-1 PMC6117613

[28] Yamamoto S , Matsumoto S-I, Goto A, Ugajin M, Nakayama M, Moriya Y, . Quantitative PCR methodology with a volume-based unit for the sophisticated cellular kinetic evaluation of chimeric antigen receptor T cells. Sci Rep 2020;10:17884. 33087808 10.1038/s41598-020-74927-8 PMC7578827

[29] Maude SL , Frey N, Shaw PA, Aplenc R, Barrett DM, Bunin NJ, . Chimeric antigen receptor T cells for sustained remissions in leukemia. N Engl J Med 2014;371:1507–17. 25317870 10.1056/NEJMoa1407222 PMC4267531

[30] Brudno JN , Somerville RPT, Shi V, Rose JJ, Halverson DC, Fowler DH, . Allogeneic T cells that express an anti-CD19 chimeric antigen receptor induce remissions of B-cell malignancies that progress after allogeneic hematopoietic stem-cell transplantation without causing graft-versus-host disease. J Clin Oncol 2016;34:1112–21. 26811520 10.1200/JCO.2015.64.5929 PMC4872017

[31] Kochenderfer JN , Somerville RPT, Lu T, Shi V, Bot A, Rossi J, . Lymphoma remissions caused by anti-CD19 chimeric antigen receptor T cells are associated with high serum interleukin-15 levels. J Clin Oncol 2017;35:1803–13. 28291388 10.1200/JCO.2016.71.3024 PMC5455597

[32] Lee DW , Kochenderfer JN, Stetler-Stevenson M, Cui YK, Delbrook C, Feldman SA, . T cells expressing CD19 chimeric antigen receptors for acute lymphoblastic leukaemia in children and young adults: a phase 1 dose-escalation trial. Lancet 2015;385:517–28. 25319501 10.1016/S0140-6736(14)61403-3 PMC7065359

[33] Mueller KT , Waldron E, Grupp SA, Levine JE, Laetsch TW, Pulsipher MA, . Clinical pharmacology of tisagenlecleucel in B-cell acute lymphoblastic leukemia. Clin Cancer Res 2018;24:6175–84. 30190371 10.1158/1078-0432.CCR-18-0758 PMC7433345

[34] Turtle CJ , Hanafi LA, Berger C, Hudecek M, Pender B, Robinson E, . Immunotherapy of non-Hodgkin's lymphoma with a defined ratio of CD8+ and CD4+ CD19-specific chimeric antigen receptor-modified T cells. Sci Transl Med 2016;8:355ra116. 10.1126/scitranslmed.aaf8621 PMC5045301 27605551

[35] Muranski P , Boni A, Wrzesinski C, Citrin DE, Rosenberg SA, Childs R, . Increased intensity lymphodepletion and adoptive immunotherapy–how far can we go? Nat Clin Pract Oncol 2006;3:668–81. 17139318 10.1038/ncponc0666 PMC1773008

[36] Yao X , Ahmadzadeh M, Lu Y-C, Liewehr DJ, Dudley ME, Liu F, . Levels of peripheral CD4(+)FoxP3(+) regulatory T cells are negatively associated with clinical response to adoptive immunotherapy of human cancer. Blood 2012;119:5688–96. 22555974 10.1182/blood-2011-10-386482 PMC3382928

[37] Ninomiya S , Narala N, Huye L, Yagyu S, Savoldo B, Dotti G, . Tumor indoleamine 2,3-dioxygenase (IDO) inhibits CD19-CAR T cells and is downregulated by lymphodepleting drugs. Blood 2015;125:3905–16. 25940712 10.1182/blood-2015-01-621474 PMC4473118

[38] Bot A , Rossi JM, Jiang Y, Navale L, Shen Y, Sherman M, . Cyclophosphamide and fludarabine conditioning chemotherapy induces a key homeostatic cytokine profile in patients prior to CAR T cell therapy. Blood 2015;126:4426.

[39] Williams KM , Hakim FT, Gress RE. T cell immune reconstitution following lymphodepletion. Semin Immunol 2007;19:318–30. 18023361 10.1016/j.smim.2007.10.004 PMC2180244

[40] Das RK , Vernau L, Grupp SA, Barrett DM. Naïve T-cell deficits at diagnosis and after chemotherapy impair cell therapy potential in pediatric cancers. Cancer Discov 2019;9:492–9. 30630850 10.1158/2159-8290.CD-18-1314 PMC6676489

[41] Gahrton G , Iacobelli S, Apperley J, Bandini G, Björkstrand B, Bladé J, . The impact of donor gender on outcome of allogeneic hematopoietic stem cell transplantation for multiple myeloma: reduced relapse risk in female to male transplants. Bone Marrow Transplant 2005;35:609–17. 15696179 10.1038/sj.bmt.1704861

[42] Gahrton G . Risk assessment in haematopoietic stem cell transplantation: impact of donor-recipient sex combination in allogeneic transplantation. Best Pract Res Clin Haematol 2007;20:219–29. 17448958 10.1016/j.beha.2006.09.007

[43] Zeier M , Döhler B, Opelz G, Ritz E. The effect of donor gender on graft survival. J Am Soc Nephrol 2002;13:2570–6. 12239247 10.1097/01.asn.0000030078.74889.69

[44] Markiewicz M , Siekiera U, Dzierzak-Mietla M, Zielinska P, Kyrcz-Krzemien S. The impact of H-Y mismatches on results of HLA-matched unrelated allogeneic hematopoietic stem cell transplantation. Transplant Proc 2010;42:3297–300. 20970675 10.1016/j.transproceed.2010.07.029

[45] Gardner RA , Finney O, Annesley C, Brakke H, Summers C, Leger K, . Intent-to-treat leukemia remission by CD19 CAR T cells of defined formulation and dose in children and young adults. Blood 2017;129:3322–31. 28408462 10.1182/blood-2017-02-769208 PMC5482103

[46] Calderon H , Mamonkin M, Guedan S. Analysis of CAR-mediated tonic signaling. Methods Mol Biol 2020;2086:223–36. 31707680 10.1007/978-1-0716-0146-4_17

[47] Gomes-Silva D , Mukherjee M, Srinivasan M, Krenciute G, Dakhova O, Zheng Y, . Tonic 4–1BB costimulation in chimeric antigen receptors impedes T cell survival and is vector-dependent. Cell Rep 2017;21:17–26. 28978471 10.1016/j.celrep.2017.09.015 PMC5645034

[48] Long AH , Haso WM, Shern JF, Wanhainen KM, Murgai M, Ingaramo M, . 4–1BB costimulation ameliorates T cell exhaustion induced by tonic signaling of chimeric antigen receptors. Nat Med 2015;21:581–90. 25939063 10.1038/nm.3838 PMC4458184

[49] Tschumi BO , Dumauthioz N, Marti B, Zhang L, Lanitis E, Irving M, . CART cells are prone to Fas- and DR5-mediated cell death. J Immunother Cancer 2018;6:71. 30005714 10.1186/s40425-018-0385-z PMC6045821

[50] Rossi J , Paczkowski P, Shen Y, Morse K, Flynn B, Kaiser A, . Preinfusion polyfunctional anti-CD19 chimeric antigen receptor T cells are associated with clinical outcomes in NHL. Blood 2018;132:804–14. 29895668 10.1182/blood-2018-01-828343 PMC6107882

[51] Derippe T , Fouliard S, Marchiq I, Dupouy S, Almena-Carrasco M, Geronimi J, . Mechanistic modeling of the interplay between host immune system, interleukin 7 and UCART19 allogeneic CAR-T cells in adult B-cell acute lymphoblastic leukemia. Cancer Research Communications 2022;2:1532–44. 10.1158/2767-9764.CRC-22-0176 PMC10036133 36970053

