# Supplementary material for: Clinical Pharmacology and Determinants of Response to UCART19, an Allogeneic Anti-CD19 CAR-T Cell Product, in Adult B-cell Acute Lymphoblastic Leukemia
Source: Cancer Res Commun. 2022 Nov 30;2(11):1520–31. doi: 10.1158/2767-9764.CRC-22-0175 (PMC10035397; doi:10.1158/2767-9764.CRC-22-0175)
Supplement: Supplementary Materials & Methods SM1 — Additional information regarding the CALM study (including study population and summary results) and UCART19 product characterization. [file crc-22-0175-s01.pdf]

SUPPLEMENTARY MATERIALS AND METHODS

CALM study population and overall results

Enrolled patients were aged between 18 and 64 years (median age = 37 years) and 44% (11) were female. Patients received 1 to 6 previous lines of therapy (median = 4) and 18 (72%) had prior allo-SCT. Twenty-two patients (88%) were lymphodepleted with FCA and 3 (12%) with FC only before being infused with UCART19. The tumor burden in BM prior to UCART19 infusion was 0 to 100 % (median blast percentage = 10%). Twenty patients (91%) over 22 for whom HLA allele typing comparison was possible displayed less than 30% HLA matches.

Overall, the safety profile of UCART19 was manageable with 6 patients (24%) displaying a grade ≥3 cytokine release syndrome and 1 patient (4%) a grade 4 neurologic toxicity. Seven patients (28%) developed grade ≥3 infections and 4 patients (16%) grade 4 prolonged cytopenia. Grade 1 acute cutaneous graft-versus-host disease occurred in 2 patients (8%). Twelve patients (48%; 95% confidence interval [CI], 28 to 69) achieved complete remission with complete or incomplete hematologic recovery as best overall response with a relapse-free survival and overall survival rate at 6 months of 55% (95% CI, 23 to 78) and 61% (95% CI, 38 to 77), respectively.

UCART19 product characterization

Final product characterization was performed on day 19 (prior to freezing) by multiparametric flow cytometry. Samples were stained with the following antibodies, combined in different panels: CD45-VB (Miltenyi Biotec Cat# 130-113-122, RRID:AB_2725950), CD45-PerCP (Miltenyi Biotec Cat# 130-113-682, RRID:AB_2726223), CD4-VB (Miltenyi Biotec Cat# 130-113-219, RRID:AB_2726030), CD4-PerCP (Miltenyi Biotec Cat# 130-113-217, RRID:AB_2726028), CD8-VB (Miltenyi Biotec Cat# 130-113-162, RRID:AB_2725990), CD8-PeCy7 (Miltenyi Biotec Cat# 130-113-159, RRID:AB_2725987), CD52-FITC (Miltenyi

Biotec Cat# 130-123-926, RRID:AB_2819543), TCRab-APC (Miltenyi Biotec Cat# 130-113-535, RRID:AB_2733457), CD62L-FITC (Miltenyi Biotec Cat# 130-113-619, RRID:AB_2733652) and CD45RA-PeVio770 (Miltenyi Biotec Cat# 130-113-919, RRID:AB_2726407). For CAR detection, either an anti-mouse IgG F(ab')2 -PE (Jackson ImmunoResearch Labs Cat# 115-116-072, RRID:AB_2338627) or the anti-idiotype antibody UCART19-PE (provided by Allogene Therapeutics) was used. For viability assessment, cells were stained with the Fixable Viability Dye eFluor® 780 (eBioscience Cat# 65-0865). Samples were analyzed in a MACSQuant (Miltenyi Biotec) using a MACSQuantify software.
