# Supplementary material for: Clinical Pharmacology and Determinants of Response to UCART19, an Allogeneic Anti-CD19 CAR-T Cell Product, in Adult B-cell Acute Lymphoblastic Leukemia
Source: Cancer Res Commun. 2022 Nov 30;2(11):1520–31. doi: 10.1158/2767-9764.CRC-22-0175 (PMC10035397; doi:10.1158/2767-9764.CRC-22-0175)
Supplement: Supplementary Figures S1-S9 — Fig.S1: Schematic diagram of CALM study design; Fig.S2: Correlation of UCART19 transgene levels evaluated by qPCR in paired peripheral blood and bone marrow aspirate samples; Fig.S3 & S4: Impact of demographic characteristics, prior therapies and tumor burden on UCART19 in vivo expansion (Cmax) or persistence (AUCTlast), respectively; Fig.S5: Impact of tumor burden at the time of UCART19 infusion on UCART19 kinetics based on response status; Fig.S6: UCART19 product characteristics; Fig.S7: Scatter plots of UCART19 cellular kinetic parameters by qPCR vs transduction efficiency and cell viability; Fig.S8: Impact of lymphodepletion on homeostatic cytokines (IL-7 and IL-15) and UCART19 cellular kinetics; Fig.S9: Impact of number of prior treatment lines on IL-7 exposure (AUC28). [file crc-22-0175-s02.pdf]

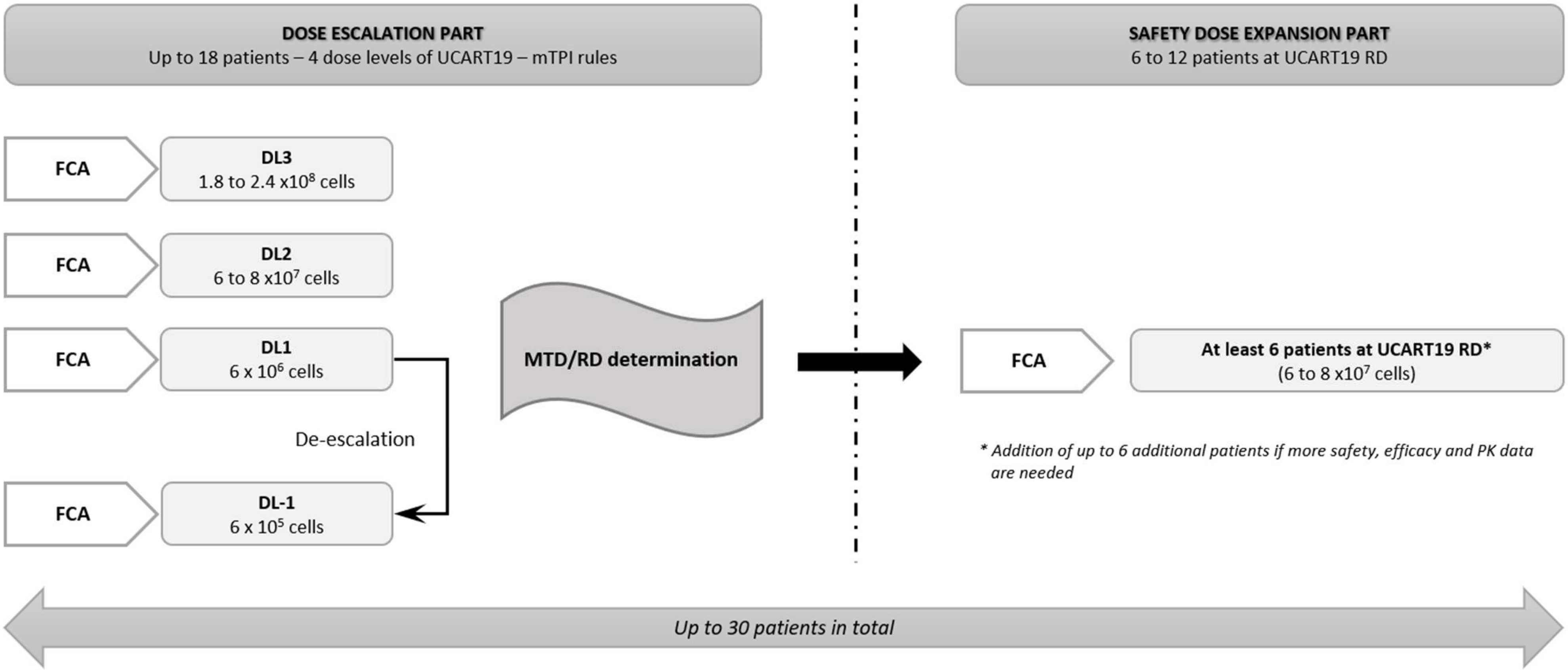

Figure S1. Schematic diagram of CALM study design.

A, alemtuzumab; C, cyclophosphamide; DL, dose level; F, fludarabine; FCA, fludarabine/cyclophosphamide/alemtuzumab lymphodepletion regimen; MTD, maximum tolerated dose; mTPI, modified toxicity probability interval; RD, recommended dose

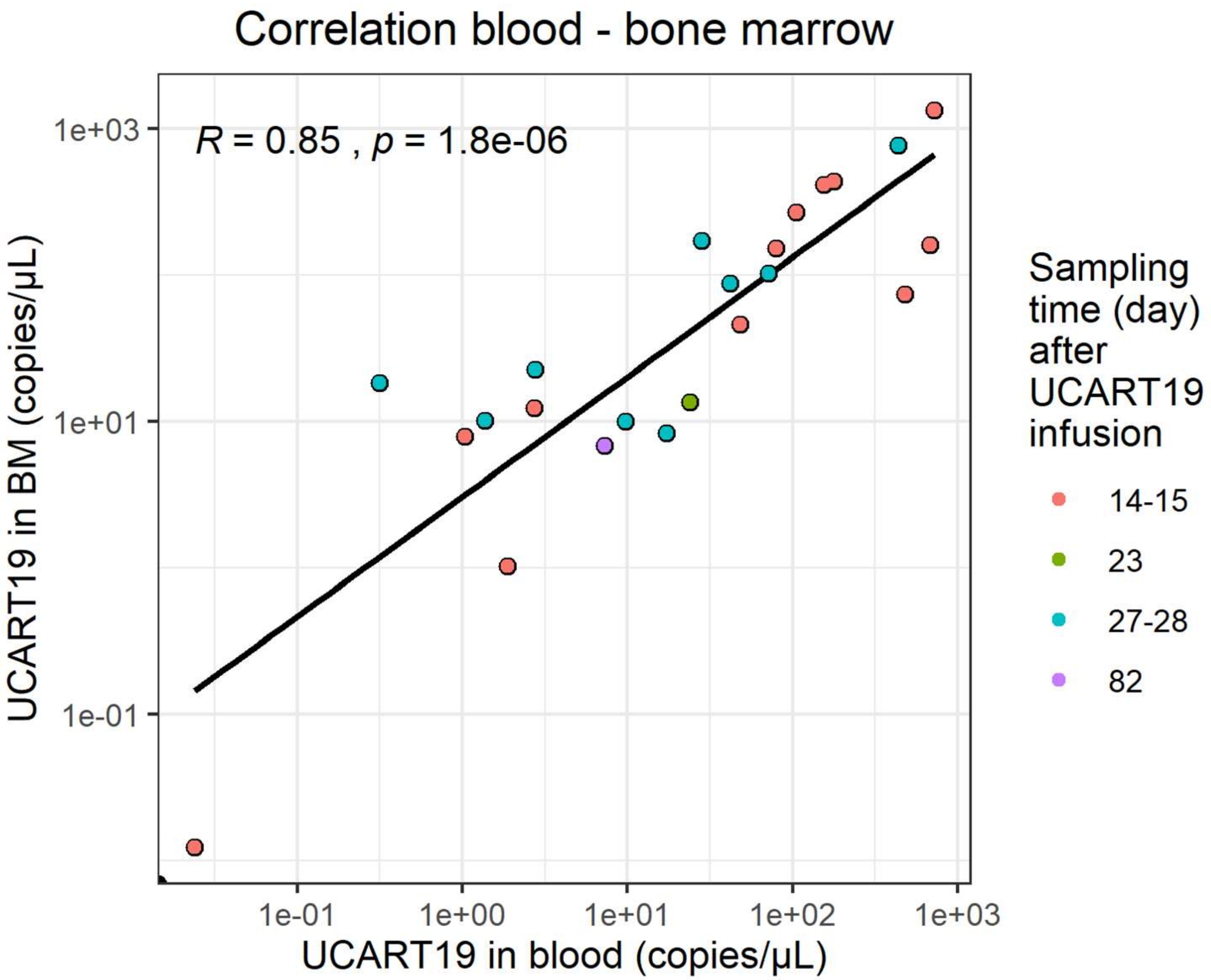

Figure S2. Correlation of UCART19 transgene levels evaluated by quantitative PCR (qPCR) in paired peripheral blood and bone marrow (BM) aspirate samples of 11 patients (23 time points) in CALM study. Colors represent different time points. Test Spearman (p=1.8 e-06).

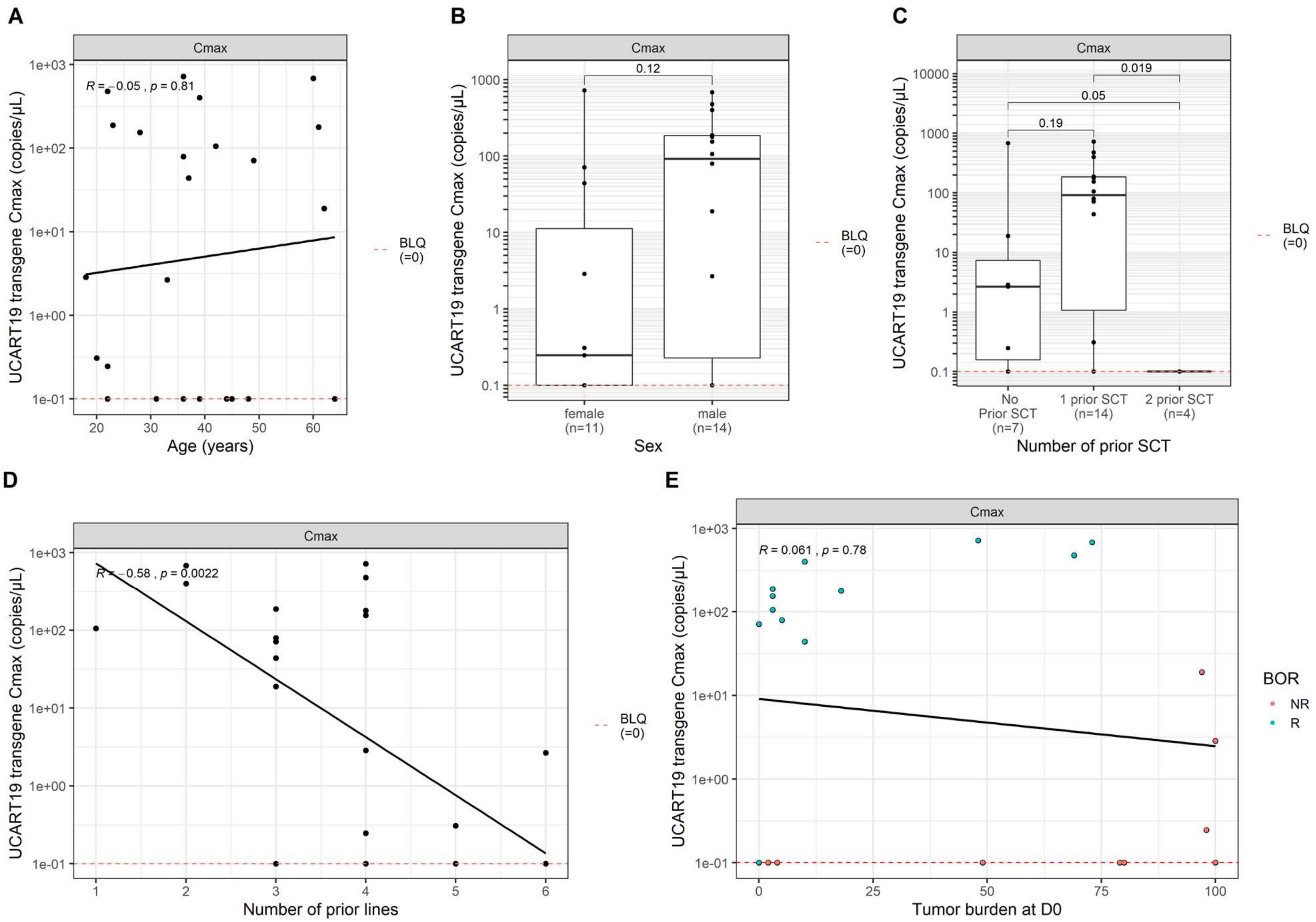

Figure S3. Impact of demographic characteristics, prior therapies and tumor burden on UCART19 in vivo expansion (Cmax). UCART19 transgene levels were evaluated by qPCR. (A) Impact of the patients' age on UCART19 Cmax. (B) Impact of the patients' gender on UCART19 Cmax. (C) Impact of the number of prior allogeneic SCT received by patients on UCART19 Cmax. (D) Correlation between number of lines of prior therapy and UCART19 Cmax. (E) Correlation between tumor burden at time of UCART19 infusion (D0) and UCART19 Cmax. Cmax, maximum peak expansion; qPCR, quantitative polymerase chain reaction; SCT, stem cell transplant.

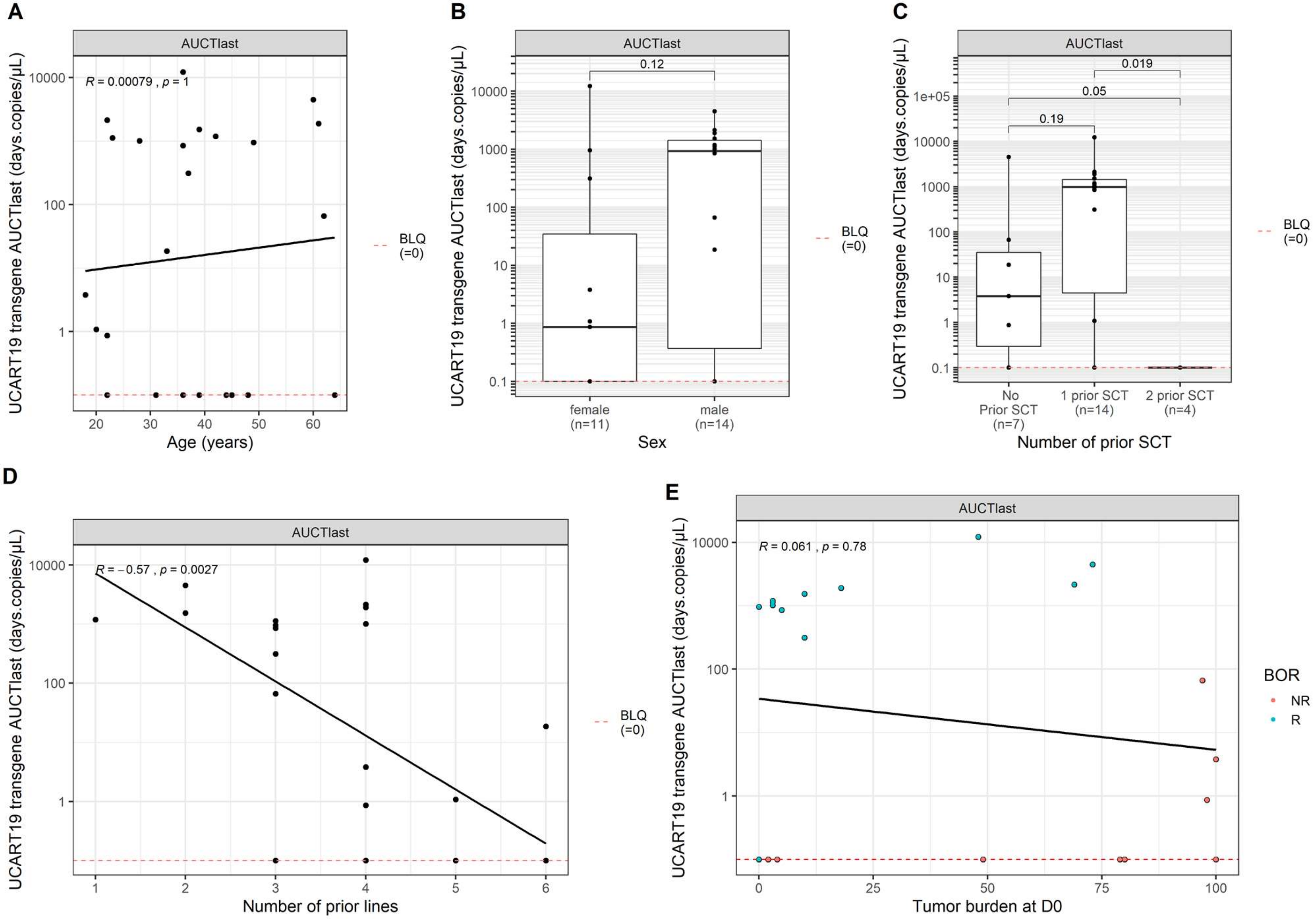

Figure S4. Impact of demographic characteristics, prior therapies and tumor burden on UCART19 persistence (AUCTlast). UCART19 trangene levels were evaluated by qPCR. (A) Impact of the patients' age on UCART19 AUCTlast. (B) Impact of the patients' gender on UCART19 AUCTlast. (C) Impact of the number of prior allogeneic SCT received by patients on UCART19 AUCTlast. (D) Correlation between number of lines of prior therapy and UCART19 AUCTlast. (E) Correlation between tumor burden at time of UCART19 infusion (D0) and UCART19 AUCTlast. AUCTlast, area under the curve from day 0 until the last observed quantifiable level of CAR transgene; qPCR, quantitative polymerase chain reaction; SCT, stem cell transplant.

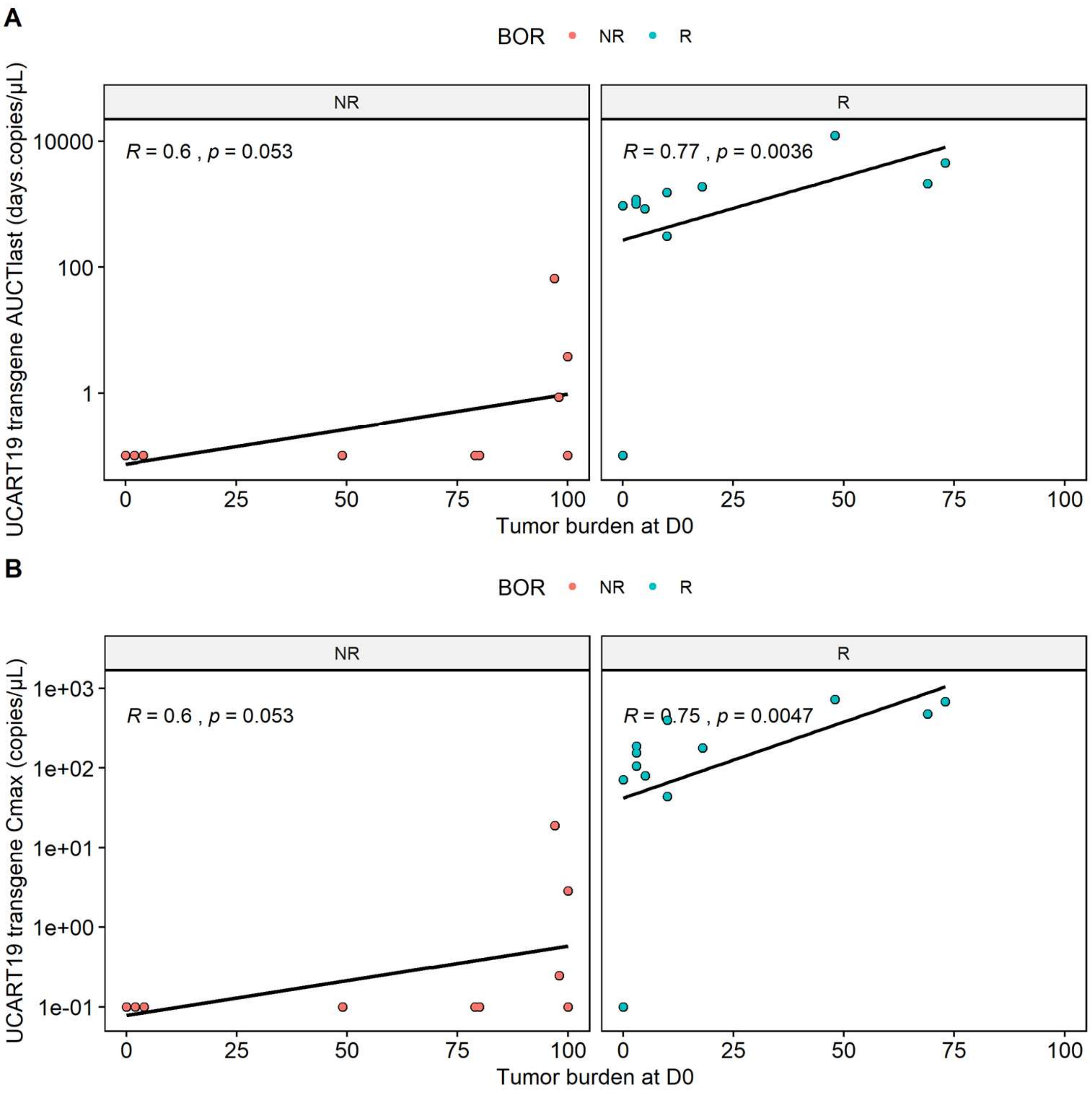

Figure S5. Impact of tumor burden at the time of UCART19 infusion on UCART19 kinetics based on response status. (A) Correlations between AUCTlast and tumor burden. (B) Correlations between Cmax and tumor burden. Positive correlations were found between tumor burden and UCART19 kinetics parameters (both AUCTlast and Cmax) when splitting the patients by Non-Responder (NR) vs Responder (R) status.

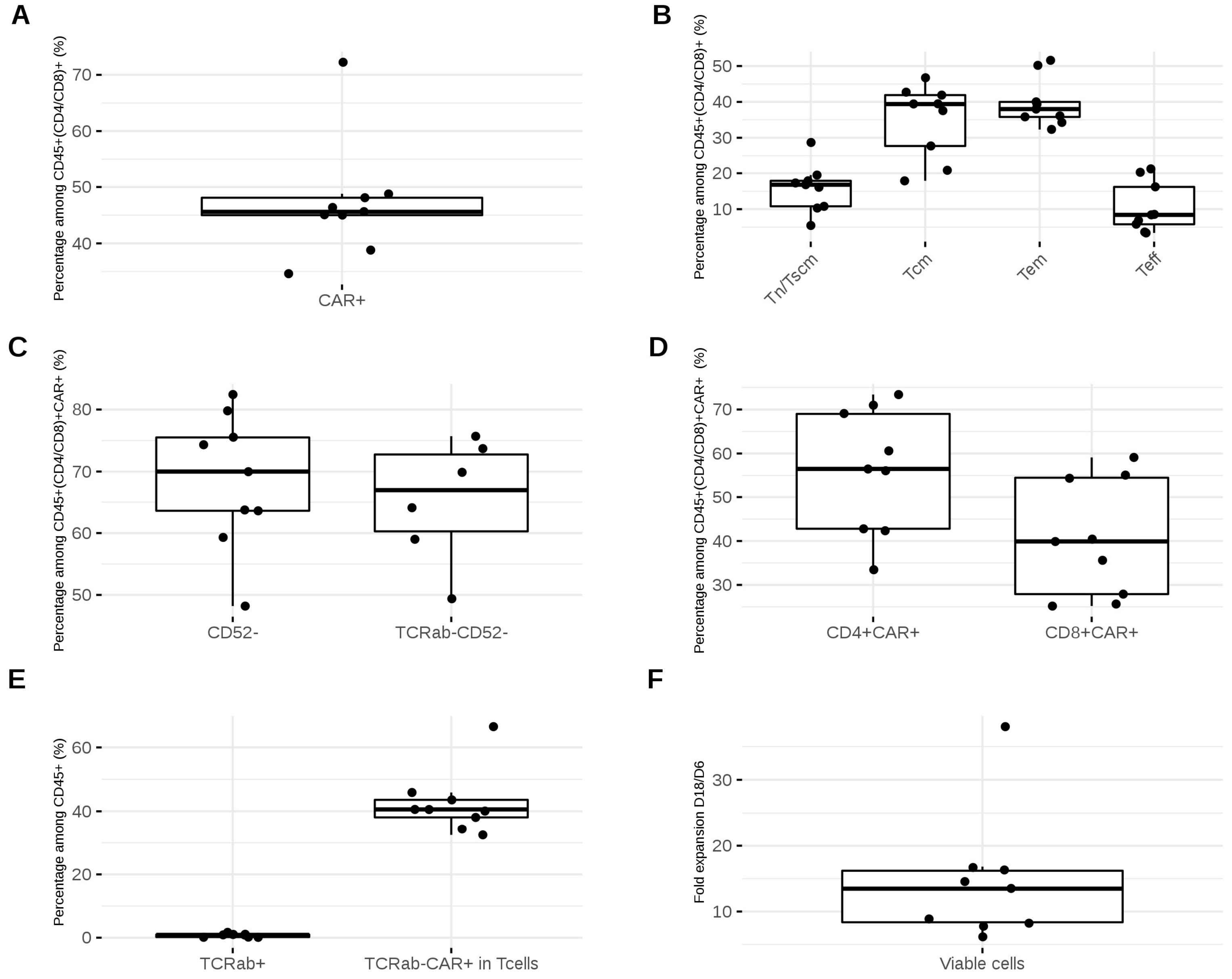

Figure S6. UCART19 product characteristics. Drug substance (product before freezing at day 19) was characterized by multiparametric flow cytometry for (A) CAR$^+$ expression and (B) memory subpopulations distribution in T cells. CD52$^-$ and TCRαβ$^-$ CD52$^-$ expression (C) and CD4$^+$ and CD8$^+$ expression (D) were assessed in CAR$^+$ T cells. (E) Total TCRαβ$^+$ and CAR$^+$ TCRαβ$^-$ T cell populations were also determined. (F) Fold expansion of total viable cells between day 6 and day 18. Expansion phase was determined such to avoid biases induced by electroporation and purification steps during the UCART19 manufacturing process. Averages and SD are shown. Tn, naïve- and Tscm, stem cell memory T cells (CD62L$^+$ CD45RA$^+$); Tcm, central memory T cells (CD62L$^+$ CD45RA$^-$); Tem, effector memory T cells (CD62L$^-$ CD45RA$^-$); Teff, effector T cells (CD62L$^-$ CD45RA$^+$).

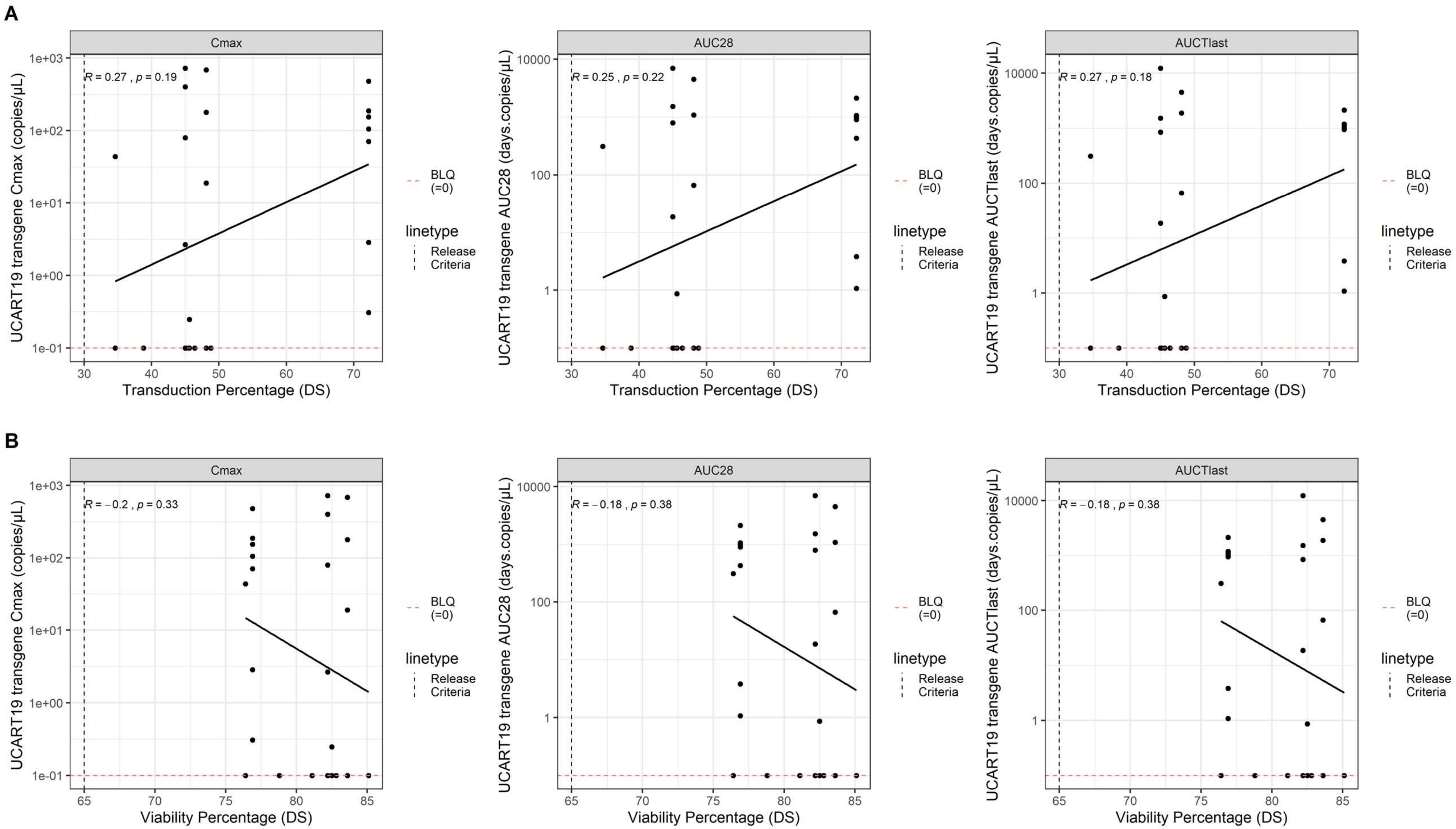

Figure S7. Scatter plots of UCART19 cellular kinetic parameters by qPCR vs transduction efficiency and cell viability. (A) Transduction efficiency. (B) Cell viability. Cmax, maximum peak expansion; AUC28, area under the curve between day 0 and day 28 post CAR T infusion; AUCTlast, area under the curve from day 0 until the last observed quantifiable level of CAR transgene. Vertical dashed lines represent criteria for GMP batch release.

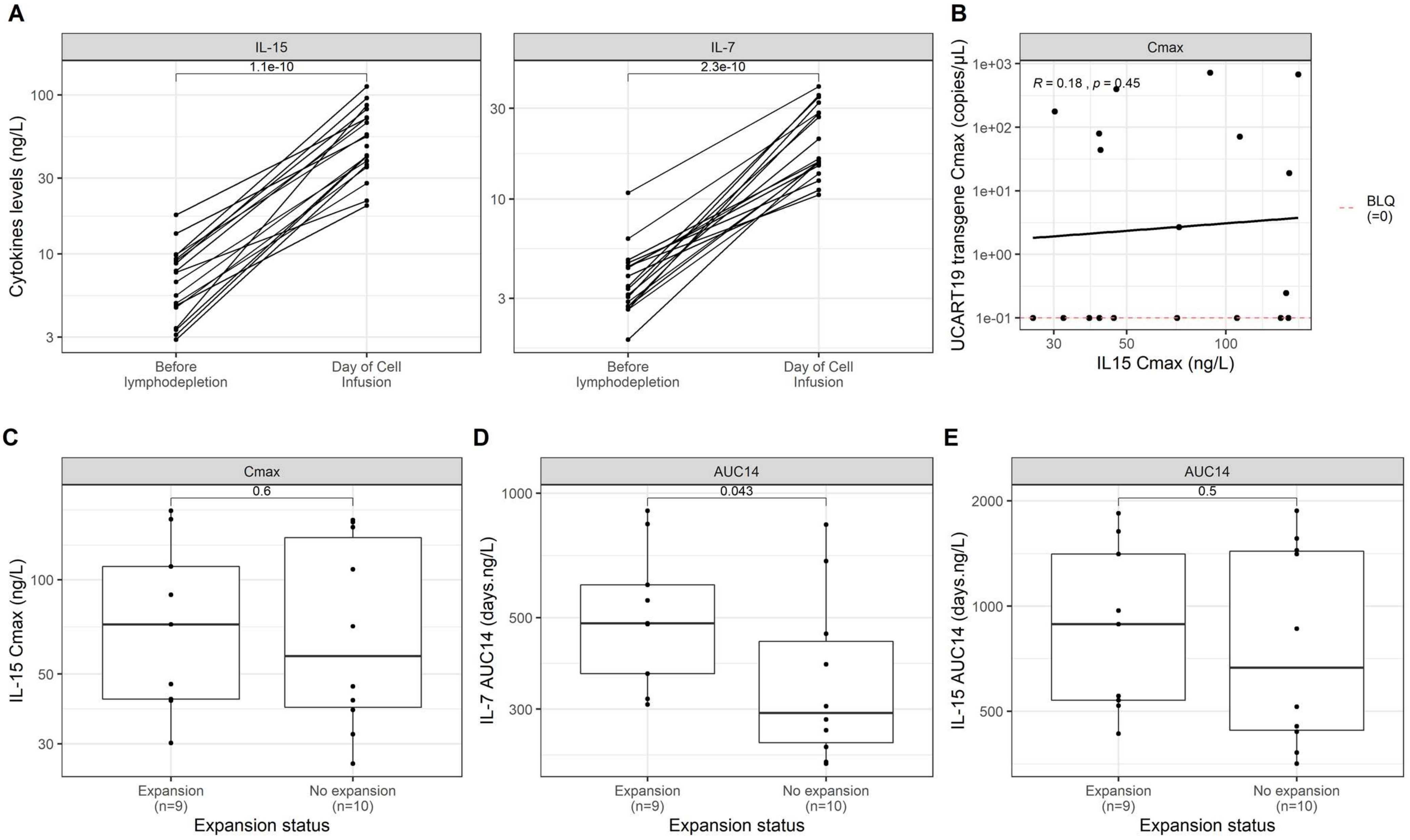

Figure S8. Impact of lymphodepletion on homeostatic cytokines (IL-7 and IL-15) and UCART19 cellular kinetics. (A) IL-15 and IL-7 levels (ng/L) in plasma samples of patients before lymphodepletion and at day of UCART19 infusion (D0), (B) absence of correlation between IL-15 peak (Cmax) and UCART19 expansion (Cmax), (C) Relationship between IL-15 Cmax and UCART19 expansion status, (D and E) correlation of IL-7 (D) and IL-15 (E) exposures from first measurement until the two first weeks following UCART19 infusion (AUC14) and UCART19 expansion.

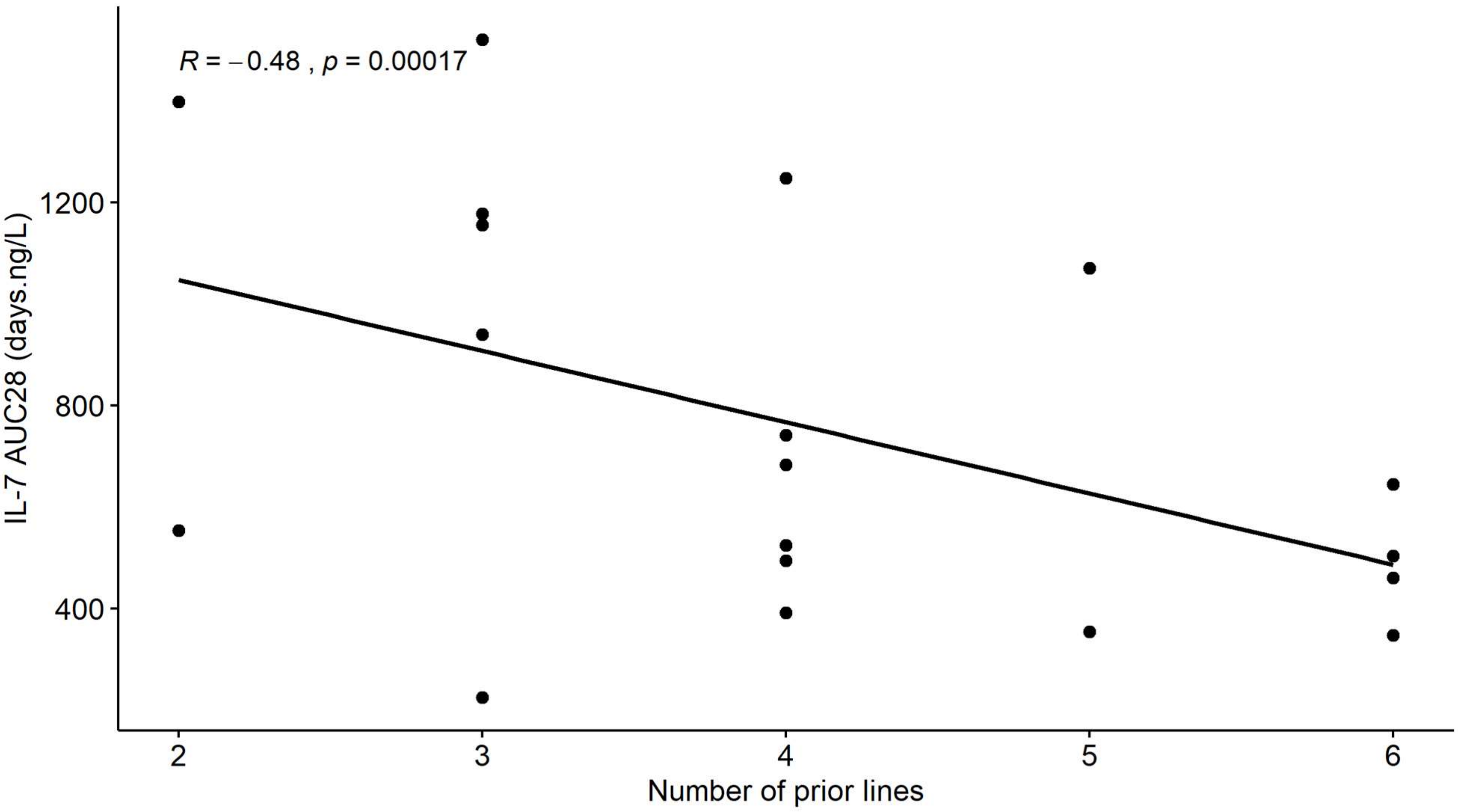

Figure S9. Impact of number of prior treatment lines on IL-7 exposure (AUC28).
