# Supplementary material for: Clinical Pharmacology and Determinants of Response to UCART19, an Allogeneic Anti-CD19 CAR-T Cell Product, in Adult B-cell Acute Lymphoblastic Leukemia
Source: Cancer Res Commun. 2022 Nov 30;2(11):1520–31. doi: 10.1158/2767-9764.CRC-22-0175 (PMC10035397; doi:10.1158/2767-9764.CRC-22-0175)
Supplement: Supplementary Tables S1-S4 — Table S1: Representativeness of CALM study participants; Table S2: UCART19 dose and cellular kinetics analysis; Table S3: Characteristics of UCART19 PBMC donors; Table S4: Impact of UCART19 and alemtuzumab doses on UCART19 expansion. [file crc-22-0175-s03.pdf]

SUPPLEMENTARY TABLES S1-S4

Cancer type	B cell acute lymphoblastic leukemia (B-ALL)
Considerations related to :	
Sex	B-ALL is a rare disease which occurs slightly more frequently in males than in females with an overall male to female ratio of 1.4.<br>In the US, in 2019, the age-standardized incidence rate was 2.1 per 100,000 in males and 1.5 per 100,000 in females. In UK, from 2016 to 2018, it was 1.3 per 100,000 in males and 0.9 per 100,000 in females.
Age	B-ALL is a disease primarily impacting children. In most countries, its incidence is approximately 3 to 4 times that in adults. Age-specific incidence rates peak in children aged 0-4 years, then drop through childhood, adolescence and young adulthood, reaching their lowest point between 30 and 50 years, then increasing gradually in adults from 50 years.
Race/ethnicity	Incidence trends of ALL demonstrate disparities by race and ethnicity. ALL usually occurs three times as frequently in Caucasians as in African-Americans. In the US, from 2000 to 2016, among persons of all ages diagnosed with ALL, approximately 35% were Latinos, 49% non-Latino Whites, 8% Asians/Pacific Islanders, 7% non-Latino Blacks, 1% American Indian/Alaska Native and 0.5% of another racial/ethnic background.
Geography	Generally, the age-standardized incidence of ALL is highest in the Americas (especially South America) and Oceania and lowest in Asia (except Singapore) and Eastern Europe (i.e., Czech Republic and Poland).<br>In the US, ALL rates vary by states and regions. In EU, rates are slightly lower in Eastern than in Western European countries.
Overall representativeness of the CALM study	B-ALL is a rare disease, especially in adults, which is the population the CALM trial was focusing on (> 18 years). The age and sex distributions within this study were similar to the average age of adult B-ALL patients with a median age of 37 years (IQR 28-45) at the time of inclusion in the CALM study and to the sex repartition with 44% of female patients included.<br>Patients from different geographical areas were enrolled, including Europe (UK and France), USA and Japan.<br>Within the 25 patients who were included in CALM, 68% were Whites, 8% were Blacks or African Americans, 8% were Asians and 16% were of another racial/ethnic background. 5% were Hispanic or Latino. Overall, the race/ethnicity distribution of the CALM study was similar to the observed incidence rate by race of ALL, except for Hispanic or Latinos who were underrepresented in this study.

Table S1. Representativeness of CALM study participants.

Data sources :

Cancer Research UK, https://www.cancerresearchuk.org/health-professional/cancer-statistics/statistics-by-cancer-type/leukaemia-all, Accessed September 2022.

Surveillance, Epidemiology, and End Results Program, https://seer.cancer.gov/statistics-network/explorer/application.html, acute lymphoblastic leukemia, Accessed September 2022.

Feng Q, De Smith AJ, Vergara-Lluri M, Muskens IS, McKean-Cowdin R, Kogan S, et al. Trends in Acute Lymphoblastic Leukemia Incidence in the United States by Race/Ethnicity From 2000 to 2016. Am J Epidemiol 2020; 190(4).

Of note, no incidence data according to sex, age, race/ethnicity or geography were provided for Europe as European Registry only provides detailed incidence data for total leukaemia and not specifically for B-ALL. IQR, Interquartile range review.

Dose UCART19	DL1 (6 e06) (n=6)	DL2 (6-8 e07) (n=12)	DL3 (1.8-2.4 e08) (n=7)	Overall (n=25)
**AUC0-28 (days.copies/µL)**				
Mean (SD)	850.3 (793.55)	780.9 (1984.39)	903.3 (1675.3)	831.8 (1624.98)
Median [Min;Max]	951.1 [1.1;2131.3]	9.7 [0;6973.8]	0 [0;4486.9]	18.6 [0;6973.8]
**AUCTlast (days.copies/µL)**				
Mean (SD)	908.5 (809.15)	1335.9 (3489.21)	904.9 (1679.19)	1112.6 (2543.48)
Median [Min;Max]	1063.5 [1.1;2133.4]	9.7 [0;12252.5]	0 [0;4497.8]	18.6 [0;12252.5]
**Cmax (copies/µL)**				
Mean (SD)	154.8 (175.9)	89.4 (206.4)	161.2 (273.21)	125.2 (214.17)
Median [Min;Max]	130.2 [0.3;477.7]	1.5 [0;721.7]	0 [0;683.5]	2.9 [0;721.7]
**Dose UCART19**	**DL1 (6 e06) (n=5)**	**DL2 (6-8 e07) (n=6)**	**DL3 (1.8-2.4 e08) (n=3)**	**Expansion (n=14)**
**Tfirst (days)**				
Mean (SD)	11.2 (3.11)	8.4 (1.99)	6.7 (0.34)	9 (2.79)
Median [Min;Max]	12 [7;14]	8.3 [6.4;11]	6.8 [6.3;6.9]	8 [6.3;14]
**Tlast (days)**				
Mean (SD)	30.8 (15.34)	36.5 (25.93)	23.1 (7.98)	31.6 (19.2)
Median [Min;Max]	28 [14;56]	35.4 [10.4;82.1]	27.7 [13.9;27.8]	28 [10.4;82.1]
**Tmax (days)**				
Mean (SD)	15.4 (3.13)	15.1 (5.97)	14.7 (5.61)	15.1 (4.65)
Median [Min;Max]	14 [14;21]	13.8 [10.4;26.8]	13.1 [10;20.9]	14 [10;26.8]

Table S2. UCART19 dose and cellular kinetics analysis.

DL, dose level; Cmax, maximum peak expansion; AUC0-28, area under the curve between day 0 to day 28 post CAR T infusion; AUCTlast, area under the curve from day 0 until the last observed quantifiable level of CAR transgene; Tfirst (samples were not collected at the same time points), time of first quantifiable UCART19 transgene; Tlast, time of last quantifiable UCART19 transgene; Tmax, time of maximal expansion.

UCART19 donor	Age	Sex	BMI	ABO	Rh
A	25	Male	28.1	AB	+
B	23	Male	22.5	O	+
C	21	Male	20.5	O	+
D	25	Male	25.7	B	+
E	23	Male	18.2	B	+
F	24	Male	28.1	A	+

Table S3. Characteristics of UCART19 PBMC donors. BMI, body mass index; ABO, ABO blood group system; Rh, rhesus blood group system.

**UCART19/alemtuzumab (dose)**	**0 mg**	**40 mg fixed**	**60 mg fixed**	**1 mg/kg [65-95 mg]**	**Total (patients)**
**DL1** (6 e06 CAR$^+$ cells)	-	0/1	-	5/5	5/6
**DL2** (6-8 e07 CAR$^+$ cells)	0/2	1/2	2/4	3/4	6/12
**DL3** (1.2-2.4 e07 CAR$^+$ cells)	0/1	3/6	-	-	3/7
**Total (patients)**	0/3	4/9	2/4	8/9	14/25

Table S4. Impact of UCART19 and alemtuzumab doses on UCART19 expansion. X/Y means X patients had an expansion among Y patients; DL, dose level.
